# NONO links circadian rhythm disruption and enhanced tumor-fibroblast crosstalk in right-sided colorectal cancer

**DOI:** 10.1186/s40364-025-00852-5

**Published:** 2025-10-31

**Authors:** Zhi-Hao Shang, Qin-Chang Zhang, Song-Yang Xi, Kai Chen, Shao-Bo Guo, Zhou Zhou, Xin-Zhuo Zhan, Yun-Xia Wu, Xin-Yi Li, Hai-Bo Cheng, Xue-Jun Song, Gui-Hua Tian

**Affiliations:** 1 Dongzhimen Hospital, Beijing University of Chinese Medicine, Beijing, China; 2 First Clinical Medical College, Nanjing University of Chinese Medicine, Nanjing, China; 3 School of Traditional Chinese Medicine, Beijing University of Chinese Medicine, Beijing, China; 4 Beijing Friendship Hospital, Capital Medical University, Beijing, China; 5 Department of Medical Neuroscience and SUSTech Center for Pain Medicine, School of Medicine, Southern University of Science and Technology, Shenzhen, China; 6 Zhenjiang Hospital of Chinese Traditional and Western Medicine, Zhenjiang, Jiangsu China

**Keywords:** Circadian rhythm, NONO, Right-sided colorectal cancer, Single-cell transcriptomics, Spatial transcriptomics

## Abstract

The biological heterogeneity between left- and right-sided colorectal cancer (CRC) poses a significant clinical challenge and the underlying regulatory mechanisms remain elusive. As an emerging hallmark of cancer, the contribution of circadian rhythm disruption (CRD) to this side-specific heterogeneity is unclear. By integrating single-cell and spatial transcriptomic analyses, this study shows that tumor cells in right-sided CRC exhibit significantly higher CRD scores and identifies the emergence of a NONO-positive tumor-cell subpopulation (NONO⁺ TC) as a key molecular feature of this phenotype. Spatial analysis further confirms that this NONO⁺ TC forms a tightly co-localized microenvironment with fibroblasts in situ. Notably, cell–cell communication analyses indicate that NONO expression does not augment the signal-sending capacity of tumor cells but instead reprograms them into highly efficient signal receivers. These augmented incoming signals predominantly originate from the more pro-tumorigenic myofibroblastic cancer-associated fibroblast (myCAF) subtype, indicating that NONO⁺ TC are particularly sensitive to malignant stromal inputs. In conclusion, our study delineates the dual role of NONO as both a circadian regulator and a pro-tumorigenic signaling hub. By enhancing tumor-cell reception of CAF-derived signals, NONO links CRD to the formation of NONO⁺ TC niches and a malignant microenvironment in right-sided CRC, providing new mechanistic insight into the spatial heterogeneity of tumors.

**Supplementary Information:**

The online version contains supplementary material available at 10.1186/s40364-025-00852-5.

Circadian rhythm refers to the physiological changes within an organism that correspond to the day-night cycle. This physiological rhythm is controlled by an endogenous biological clock and regulated by external light-dark cycles [1, 2]. Disruption of circadian rhythms can lead to various health issues, including sleep disorders, metabolic disturbances, cardiovascular diseases, and cancer [3]. In modern society, circadian rhythm disruption has become increasingly prevalent due to shift work, trans-meridian travel, and irregular lifestyles [4, 5]. Non-POU domain-containing octamer-binding protein (NONO) occupies a pivotal position in the mammalian circadian network. NONO forms a direct complex with the core negative-feedback components PER1 and PER2; accordingly, RNAi-mediated depletion of NONO dampens intrinsic cellular oscillations and renders animals nearly arrhythmic [6]. A recent review linking NONO, circadian regulation, and cancer notes that NONO overexpression or dysfunction is a common feature across multiple tumour types, and its central role in the clock–cell-cycle–metabolism axis positions NONO as a promising anticancer target [7].

The relationship between circadian rhythm disruption and tumor development is complex and intimate, involving multiple interacting mechanisms. Studies have shown that abnormal expression of key clock genes, such as Per2, may weaken its tumor suppressor function [8]. Additionally, the influence of the clock protein BMAL1 on the cell cycle regulatory protein Wee1 can lead to cell cycle dysregulation, increasing genomic instability [9]. Concurrently, melatonin secretion abnormalities caused by circadian rhythm disruption may suppress NK cell activity, weakening immune surveillance [10]. Furthermore, changes in hormonal secretion patterns, such as cortisol, can affect cell proliferation and apoptosis processes by regulating the expression of genes like Bcl-2 and BAX [11]. These mechanisms interact to form a complex network through which circadian rhythm disruption promotes tumor development, highlighting the potential importance of maintaining normal circadian rhythms in the prevention and treatment of tumors.

Colorectal cancer (CRC) is one of the most common malignant tumors globally, with increasing incidence and mortality rates in many countries. CRC can be classified into left-sided and right-sided colorectal cancer based on anatomical location, with significant differences in clinical presentation, pathological features, and prognosis [12, 13]. Left-sided colorectal cancer typically occurs in the descending and sigmoid colon, presenting as annular strictures and bleeding, with a shorter disease course and more apparent early symptoms. By contrast, right-sided colorectal cancer arises in the cecum and ascending colon; it is more common in older patients and typically presents with anemia and abdominal discomfort, with a longer disease course and less conspicuous early symptoms [14, 15]. Moreover, left-sided and right-sided colorectal cancers differ significantly in molecular characteristics, gene mutations, microenvironment, and metabolic pathways. For instance, right-sided colorectal cancer is often associated with microsatellite instability (MSI) and BRAF mutations, while left-sided colorectal cancer more commonly harbors KRAS and TP53 mutations. Additionally, the tumor microenvironment of right-sided colorectal cancer exhibits more prominent inflammatory responses and immune evasion mechanisms, and its metabolic pathways also display significant regional specificity [16, 17].

Single-cell transcriptomics can precisely analyze gene expression in individual cells, thereby revealing cellular heterogeneity and the functions of specific cell subpopulations [18]. Spatial transcriptomics, on the other hand, preserves spatial information of tissues, demonstrating the distribution patterns of gene expression within tissue structures [19, 20]. The combination of these two approaches not only provides detailed gene expression data at the cellular level but also maps this data back to the spatial context of the tissue, enabling a more comprehensive understanding of tissue complexity and cell-cell interactions. This integrated approach holds significant importance for studying disease microenvironments, tissue development, and regeneration.

Integrative analyses of single-cell and spatial transcriptomic datasets describe circadian features across colorectal tumor laterality, with particular attention to NONO-positive tumor cells(NONO⁺ TC) in right-sided disease.We observed markedly higher circadian-disruption scores in right-sided CRC, with NONO positivity emerging as a defining molecular feature. Spatial mapping and multiscale modeling revealed robust co-localization and bidirectional coupling between NONO⁺ TC and fibroblasts. CellChat and receptor–ligand analysis identified TENASCIN and THBS as dominant stromal signaling pathways, with fibroblasts acting as primary signal senders and NONO⁺ TC as efficient receivers. Pseudotime and functional assays further supported a mechanism whereby NONO modulates core clock genes and cell-cycle regulators and impacts tumor proliferation and migration. Collectively, we propose a NONO-centric framework that links circadian disruption to a malignant microenvironment in right-sided CRC, offering a rationale for side-specific stratification and potential therapeutic targeting.

## Materials and methods

### Datasets & Materials

The bulk RNA sequencing data and clinicopathological characteristics for TCGA-CRC were downloaded from the UCSC Xena website (https://xena.ucsc.edu/). The cohort includes 512 colon cancer and 177 rectal cancer samples, with all expression profile data presented in FPKM format. Batch correction and cross-cohort integration were performed using limma (v3.54.2) and sva (v3.46.0). Single-cell RNA sequencing (scRNA-seq) data for colorectal cancer were obtained from GSE200997, comprising 16 samples, with 8 from left-sided and 8 from right-sided colorectal cancer. Spatial transcriptomics RNA sequencing (stRNA-seq) data for primary colorectal cancer were sourced from the single-cell colorectal cancer liver metastasis (CRLM) atlas portal (http://www.cancerdiversity.asia/scCRLM). The gene set for circadian rhythm (GO:0007623) was obtained from the Molecular Signatures Database (https://www.gsea-msigdb.org/gsea/msigdb). GSE39582 was used as a validation cohort for prognostic analysis. Antibodies were purchased from Abmart Pharmaceuticals (Shanghai), including NONO antibody (T58525S), EpCAM antibody (TN24218S), and Vimentin antibody (T55134S). siRNA sequences targeting human NONO were obtained from Tsingke Biotechnology Co., Ltd. (Shanghai, China) and are listed in Supplementary Table 5. Patient tissue samples were provided by Jiangsu Provincial Hospital of Traditional Chinese Medicine, with clinical ethics approval number: 2023NL-003-02.

### Quality control and dimensionality reduction clustering 

The raw gene expression matrices were imported using the Read10X function from the Seurat(version 4.3.0) package in R. For each sample, a Seurat object was instantiated via the CreateSeuratObject function, with the project parameter assigned the respective sample name. Quality control metrics were computed for each cell, including the percentages of mitochondrial genes (percent.mt) and hemoglobin genes (percent.HB). The PercentageFeatureSet function was employed to calculate percent.mt with the pattern set to “^MT-“, while percent.HB was derived using a predefined list of hemoglobin genes. Violin plots were constructed to depict the distribution of quality control metrics before and after filtering for each sample. Cells were filtered based on the following criteria: detection of 200 to 4000 genes and percent.mt less than 10%. The filtered Seurat objects were normalized using the LogNormalize method with a scale factor of 10,000. Variable features were identified using the FindVariableFeatures function, applying the “vst” method to select the top 2000 variable features. Data scaling was performed with the ScaleData function, followed by principal component analysis (PCA) using the RunPCA function. The results of the dimensionality reduction were visualized with the DimPlot function, with cells colored according to their original sample identifiers.

### Circadian rhythm disruption scoring

We integrated five analytical methods to evaluate the differential scoring of circadian rhythms among cells. First, we used the AUCell_buildRankings function to rank the gene expression matrix, and then calculated the AUC values for gene sets using the AUCell_calcAUC function, setting aucMaxRank to 10% of the number of rows in the ranking matrix. UCell and singscore scoring were performed using the irGSEA.score function from the irGSEA(Version 3.3.2) package, with parameters including assay set to RNA, slot set to data, random seed set to 123, number of cores set to 1, and using custom gene sets (geneset). The analysis methods were UCell and singscore, with the kernel density estimation function set to Gaussian. The results were converted to data frames, and the count matrices for UCell and singscore were extracted separately and transposed.Gene Set Variation Analysis (GSVA) was conducted using the GSVA (Version 1.46.0)package. First, the scRNA-seq data was converted to matrix form, then analyzed using the gsva function, with parameters including the kernel density estimation function set to Gaussian, method set to ssgsea, and absolute ranking set to True. Finally, gene set scores were added to the Seurat object using the AddModuleScore function. The scoring results from different methods were combined, normalized, and scaled to 0–1. The total score was calculated and added to the metadata of the scRNA object. Results were visualized using DotPlot, VlnPlot, and FeaturePlot functions, and significance analysis was performed using the ggviolin function. Pearson and Spearman correlation analyses were performed in R to assess the association between tumor-cell NONO expression and circadian rhythm disruption (CRD) scores.

### Infercnv

To infer copy number variations (CNVs) from single-cell transcriptome data, we employed the InferCNV method. First, we loaded the pre-processed single-cell RNA sequencing data and extracted the raw counts matrix. Next, we downloaded the genome annotation file (hg38_gencode_v27.txt) and extracted the genes common to both the annotation file and the counts matrix by comparing their respective gene lists. We saved this annotation information as a text file (celltype.label.txt) for use in the InferCNV analysis.Subsequently, we created an inferCNV object using the CreateInfercnvObject function and performed the inferCNV analysis using the run function.

### Pseudotime analysis

We performed pseudotime analysis on scRNA-seq data, using Seurat for preprocessing and Monocle (v2.26.0) for trajectory inference. Initially, we loaded the Seurat object and extracted a subset comprising tumor cells. The gene expression matrix was then converted to a sparse matrix format. Using the Monocle(Version 2.26.0) package, we created a CellDataSet object, and estimated size factors and dispersions. High-dispersion genes were identified using the dispersionTable function and employed for cell ordering. Dimensionality reduction was executed using the DDRTree algorithm, followed by cell ordering based on a predefined root state. Key differentially expressed genes were visualized through a pseudotime heatmap. State trajectory and pseudotime plots were generated and subsequently combined. Additionally, the DimPlot function was utilized to visualize cells grouped by seurat clusters and segmented by tissue type.

### Cell-cell communication analysis

Cell–cell communication was analyzed with CellChat (v1.6.1) in R. We loaded the Seurat object and stratified tumor cells by NONO expression into NONO⁺ TC and NONO^−^TC. Tumor cells were further stratified by CRD score into HCR TC and LCR TC, while other cell types retained their original annotations. Gene expression matrices and metadata were extracted from the Seurat object as input for CellChat analysis. The CellChatDB.human database was loaded, focusing on the “Secreted Signaling” subset. Overexpressed genes and ligand-receptor pairs were identified using the identifyOverExpressedGenes and identifyOverExpressedInteractions functions, respectively. Ligands and receptors were projected onto the human protein-protein interaction (PPI) network. Communication probabilities between cell groups were calculated using the computeCommunProb function. Cell-cell communications involving fewer than 10 cells were filtered out using the filterCommunication function. Pathway-level communication probabilities were computed using the computeCommunProbPathway function. Cell-cell communication networks were subset and aggregated using the subsetCommunication and aggregateNet functions. NetVisual circle was used to visualize interaction counts and strengths, with emphasis on those originating from the NONO⁺ TC group. Additionally, bubble plots were generated using the netVisual bubble function to visualize communication patterns between the NONO^−^ TC cell group and other cell groups.

### Metabolic assessment

The activity of metabolic pathways within each spatial spot was quantified using the scMetabolism R package (v0.2.1). Specifically, we executed the sc.metabolism. Seurat function on the Seurat object, which internally employs the “AUCell” algorithm. This rank-based method robustly calculates an enrichment score for a given gene set on a per-spot basis. For our analysis, we used the comprehensive collection of metabolic pathways from the Kyoto Encyclopedia of Genes and Genomes (KEGG) database as the reference gene sets, and the imputation parameter was set to FALSE. This workflow yielded a quantitative matrix of metabolic activity scores for each spot across the tissue slice.

### Spatial transcriptomic data quality control and dimensionality reduction clustering

Spatial transcriptomic data were subjected to processing and analysis utilizing the Seurat package. Raw data acquisition was accomplished via the Load10X_Spatial function, with specific data directory and file nomenclature parameters. Quality control metrics, encompassing the number of genes detected per spot (nCount Spatial) and the proportion of mitochondrial genes (percent.mt), were visualized through violin plots and spatial feature plots. Ribosomal genes (denoted by prefixes “RPS,” “RPL,” and “MRP”) and mitochondrial genes (prefixed with “MT-“) were excluded from the dataset. To mitigate noise, genes expressed in fewer than 10 spots were eliminated. Data normalization was performed using the SCTransform function, which stabilizes variance across diverse gene expression levels. Principal component analysis (PCA) was conducted on the normalized data utilizing the RunPCA function. The number of significant principal components was ascertained through examination of the elbow plot. Clustering was executed using the FindNeighbors and FindClusters functions, with dimensionality specified based on the elbow plot analysis. Cluster results were visualized via the DimPlot and SpatialDimPlot functions to assess cluster integrity and spatial distribution. Cluster-specific marker gene expression was depicted using the DotPlot function, with marker genes derived from a pre-generated dataframe (df2). Plots were customized for readability (rotated x-axis labels, color gradients, and right-aligned legends).

### Spatial transcriptomic data Deconvolution

Spatial transcriptomic data were deconvolved using Robust Cell Type Decomposition (RCTD) (spacexr v2.2.1). Single-cell RNA sequencing (scRNA-seq) data were processed and annotated employing the Seurat package, with subsequent filtration to retain only cells derived from colorectal cancer tissues. Tumor cells were further stratified by circadian-rhythm disruption (CRD) into high-CRD and low-CRD subpopulations. A reference object was constructed from the scRNA-seq count matrix and corresponding cell type annotations using the Reference function in spacexr. Spatial transcriptomic data were processed via the SpatialRNA function, incorporating tissue coordinates and count matrices. The RCTD model was generated using the create. RCTD function, specifying the spatial data (puck) and reference scRNA-seq data as inputs. Deconvolution was executed using the run.RCTD function, with the doublet mode parameter set to “doublet.” Deconvolved cell type proportions and annotations were extracted from the RCTD model results. These annotations were subsequently integrated into the Seurat object containing the spatial transcriptomic data. Visualization of the spatial distribution of deconvolved cell types was achieved using the SpatialDimPlot function, with custom color schemes assigned to each cell type to enhance visual interpretation.

### Spatial interaction analysis

Spatial interactions among cell types were investigated utilizing the mistyR(Version 1.6.1) package. The deconvolved cell type proportions and spatial transcriptomic data served as input for the analysis. A custom function, run colocalization, was defined to execute MISTy (Multiscale Spatial Interaction Module) for each spatial context (intra, juxta, and para) and generate corresponding interaction results. MISTy analysis was conducted using the run misty seurat function, with parameters specifying spatial data, detection information, cell type features, and spatial context parameters. The interaction results were subsequently aggregated using the collect results function, facilitating comprehensive interpretation of the spatial cellular interactions within the tissue microenvironment.

### Cell-cell interaction analysis

Intercellular communication networks were inferred from ligand–receptor analysis using the run cci function in stlearn. The analysis encompassed spots exhibiting a minimum of 5 expressed ligand-receptor pairs, with a cell proportion threshold of 0.1 applied to ascertain cellular presence within each spatial location. Statistically significant ligand-receptor interactions were employed for predictive modeling, incorporating 50 permutations for robust statistical evaluation. The resultant cell-cell interaction landscape was visually rendered through a suite of functions, including lr cci map for ligand-receptor spatial mapping, cci map for overall interaction intensity visualization, ccinet plot for network representation, and lr chord plot for circular visualization of ligand-receptor connections. This multi-faceted approach provided a comprehensive depiction of the spatial cellular interactome within the tissue microenvironment.

### Multiple Immunofluorescence and immunohistochemistry

We performed multiplex immunofluorescence on tumor tissues from patients at Jiangsu Province Hospital of Traditional Chinese Medicine using antibodies against NONO (T58525S), EpCAM (TN24218S), and Vimentin (T55134S); nuclei were counterstained with DAPI. Initially, tumor tissues were fixed in 4% paraformaldehyde at 4 °C for 24 h and embedded in paraffin. Tissue sections were deparaffinized in xylene, rehydrated through a graded ethanol series, and subjected to antigen retrieval in citrate buffer (pH 6.0) using microwave heating.The sections were then blocked with 5% normal goat serum in PBS and incubated overnight at 4 °C with a mixture of primary antibodies diluted in 1% BSA. Subsequently, the sections were incubated with species-specific fluorescent secondary antibodies for 1 h at room temperature in the dark. Nuclear staining was performed using DAPI at a concentration of 1 µg/mL. Finally, the sections were mounted with fluorescent mounting medium and imaged using a fluorescence microscope equipped with appropriate filter sets.

For immunohistochemistry, tissues were fixed, paraffin-embedded, and sectioned. Deparaffinization and rehydration were performed using xylene and a graded ethanol series. Antigen retrieval was conducted using an appropriate buffer solution and heat treatment. Endogenous peroxidase activity and non-specific binding sites were blocked, followed by incubation with primary and secondary antibodies. 3,3’-Diaminobenzidine (DAB) was used for chromogenic detection, and hematoxylin was applied for counterstaining. The sections were subsequently dehydrated, cleared, and mounted. Results were observed and photographed using a light microscope.

### Univariate and multivariate COX regression analysis

In this study, we initially conducted univariate COX regression analysis on all selected genes to identify those significantly associated with patient survival. We employed the coxph function from the survival(Version 3.4.0) package, using survival time (futime) and survival status (fustat) as the dependent variables, and gene expression levels as the independent variables. Genes with a p-value less than 0.05 were considered significantly associated with survival. For these significant genes, we calculated their hazard ratios (HR) and 95% confidence intervals. We then fit a multivariable Cox model to evaluate the independent effects of these genes on prognosis. The model was further refined using stepwise regression to select the optimal combination of variables.

### LASSO regression analysis

To further narrow down the most predictive genes from a large pool of candidates, we applied LASSO (Least Absolute Shrinkage and Selection Operator) regression analysis. Using the glmnet(Version 4.1.8) package, we performed LASSO regression on the significant genes identified from the univariate analysis and selected the optimal penalty parameter (λ) through 10-fold cross-validation. LASSO regression is effective in handling high-dimensional data by retaining variables with predictive value while eliminating redundant or irrelevant variables. Ultimately, we extracted genes with non-zero regression coefficients, considering these as key contributors to the survival prediction model.

### Survival curve and ROC curve analysis

To evaluate model performance, we generated Kaplan–Meier survival curves and time-dependent ROC curves. Patients were stratified into high- and low-risk groups by risk score; survival curves were generated with survfit and ggsurvplot to compare outcomes between groups. Additionally, we employed the timeROC(Version 0.4) package to calculate AUC values at 1, 2, and 5 years, assessing the model’s predictive accuracy at different time points.

### Cell culture and siRNA transfection

Human colorectal cancer cell lines HCT 116 and RKO were obtained from the Cell Bank of the Chinese Academy of Sciences. Cells were maintained in RPMI-1640 (Gibco, C11875500BT). The medium was supplemented with 10% (v/v) FBS and 1% (v/v) penicillin–streptomycin (Gibco). Small interfering RNA (siRNA) transfection was performed on both HCT-116 and RKO cells. Three distinct siRNA sequences targeting NONO were designed and evaluated. The sense strand sequences were as follows: siNONO-1, 5’-GGACCAGUUAGAUGAUGAA-3’; siNONO-2, 5’-GGCUGUAGUCAUUGUGGAU-3’; and siNONO-3, 5’-GGAAGAGCUGCACAACCAA-3’ (Table S5). Based on knockdown efficiency, siNONO-1 was selected for subsequent functional experiments (sFig 7).

### Cell proliferation assay (CCK-8)

Cell proliferation was assessed using the Cell Counting Kit-8 (CCK-8) (Beyotime, Shanghai, China, Cat# C0038). After cell attachment, proliferation was measured at 24 h. 10 µL of CCK-8 solution was added to each well, followed by incubation for 2 h at 37 °C. The absorbance at 450 nm was then measured using a microplate reader (BioTek, USA).

### Wound healing assay

Cell migration ability was evaluated using a wound healing assay. Cells were seeded into 6-well plates and cultured until they reached 90–100% confluence. A sterile 10 µL pipette tip was used to create a linear scratch (wound) in the center of the cell monolayer. The detached cells were washed away with phosphate-buffered saline (PBS). The plates were then replenished with serum-free medium and cultured. Images of the wound area were captured at 0 h and 24 h post-scratching using an inverted microscope. The wound closure rate was quantified using ImageJ software. The closure rate was calculated as: [(Initial Wound Area - Final Wound Area) / Initial Wound Area] × 100%.

### EdU (5-ethynyl-2’-deoxyuridine) incorporation assay

DNA synthesis and cell proliferation were further detected using a BeyoClick™ EdU Cell Proliferation Kit with Alexa Fluor 488 (Beyotime, Shanghai, China, Cat# C0071S) according to the manufacturer’s instructions. RKO and HCT 116 cells were seeded into 6-well plates containing sterile coverslips and cultured for 24 h. The cells were then incubated with 50 µM EdU for 2 h. Subsequently, the cells were fixed with 4% paraformaldehyde for 15 min and permeabilized with 0.5% Triton X-100 for 10 min. After washing with PBS, the cells were exposed to the Click-iT reaction cocktail for 30 min in the dark. Finally, the cell nuclei were counterstained with Hoechst 33,342 for 10 min. Images were captured using a fluorescence microscope, and the percentage of EdU-positive cells (proliferating cells, green fluorescence) relative to the total number of Hoechst-stained cells (total cells, blue fluorescence) was calculated.

### Quantitative real-time PCR (qRT-PCR)

Total RNA was extracted from RKO and HCT 116 cells using TRIzol Reagent according to the manufacturer’s protocol. For reverse transcription, 1 µg of total RNA was converted to cDNA using the Evo M-MLV RT Kit with gDNA Clean for qPCR (Accurate Biology, Hunan, China, Cat# AG11705) following the manufacturer’s instructions. Quantitative real-time PCR was performed using the SYBR^®^ Green Premix Pro Taq HS qPCR Kit (Accurate Biology, Hunan, China, Cat# AG11701) on an ABI 7500 Real-Time PCR System (Applied Biosystems, USA). The 20 µL reaction mixture contained 10 µL of 2× SYBR^®^ Green Premix, 0.8 µL of each forward and reverse primer (10 µM), 2 µL of cDNA template, and was brought to a final volume with nuclease-free water. The thermal cycling conditions were as follows: initial denaturation at 95 °C for 30 s, followed by 40 cycles of 95 °C for 5 s and 60 °C for 30 s. A melting curve analysis was performed after amplification to confirm product specificity.

## Results

### Single-cell sequencing atlas of left- and right-sided colorectal cancer

**Fig. 1:**
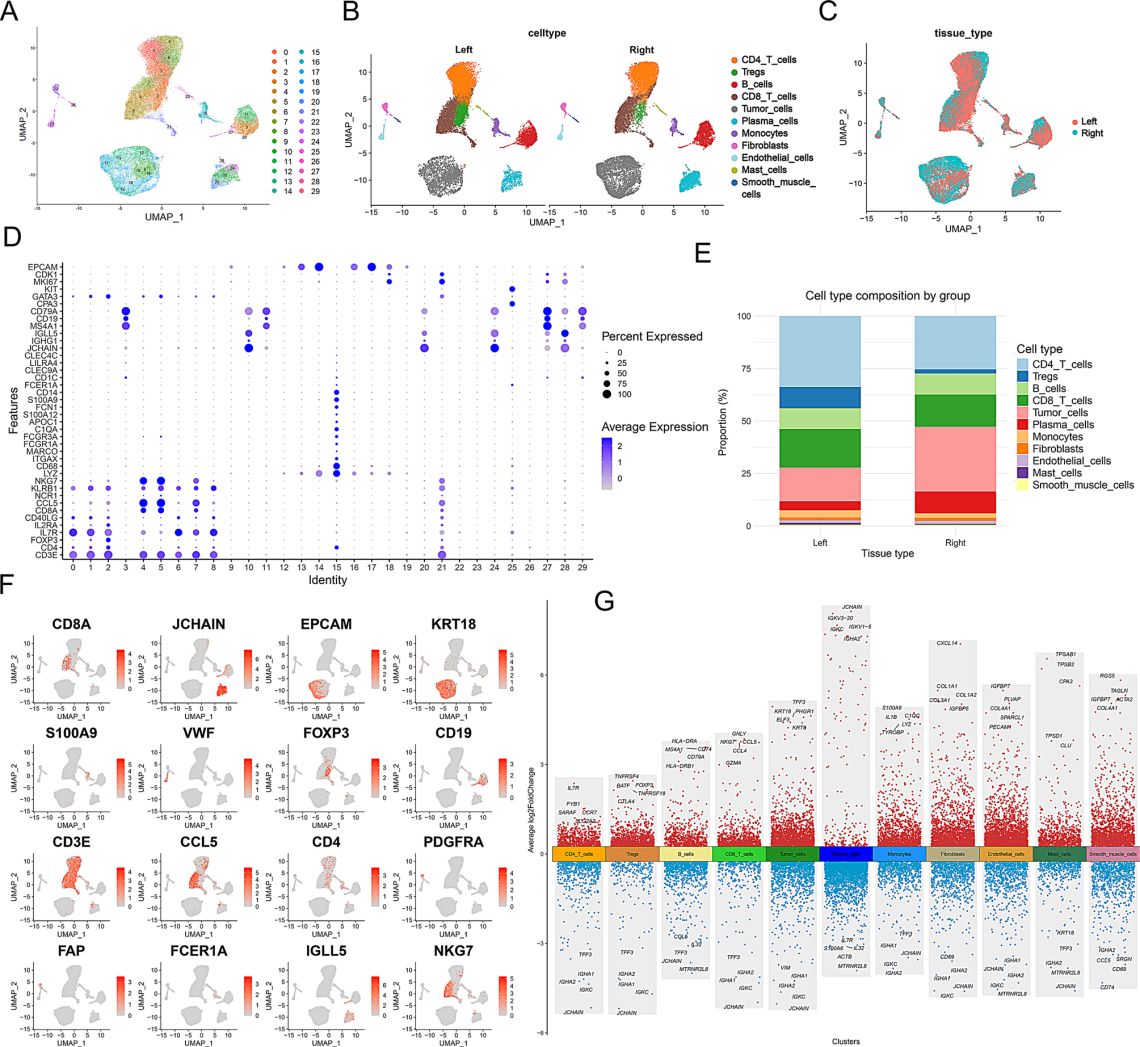
Single-cell transcriptomic atlas of left and right colorectal cancer. (**A**) UMAP plot showing the distribution of different cells, with each color representing a cell type. The x-axis and y-axis represent the two main UMAP components. (**B**) UMAP plot displaying the distribution of identified cell types in left and right colorectal cancer. (**C**) UMAP plot illustrating the distribution of cells in left and right colorectal cancer, with different colors representing different tissue types. (**D**) Dot plot showing the expression of marker genes in various cell populations. The size of the dots represents the percentage of gene expression, while the color intensity indicates the average expression level. (**E**) Bar graph comparing the proportions of different cell types in left and right tissues. Red represents right-sided tissue, and blue represents left-sided tissue. (**F**) Multiple UMAP plots showing the expression distribution of specific genes across different cell populations. The color intensity indicates the level of gene expression. (**G**) Advanced volcano plot illustrating differential gene expression across different cell populations. The y-axis represents the log2 fold change in gene expression, and the x-axis shows the different cell subgroups. Red dots in the upper half indicate significantly upregulated genes, and blue dots in the lower half indicate significantly downregulated genes

We first performed dimensionality reduction clustering on single-cell sequencing data from tissue samples of 8 left-sided and 8 right-sided colorectal cancer patients (sFig. 1A and Fig. 1A). Cell subpopulations were identified and annotated based on marker genes and their distribution in UMAP plots (sFig. 1B, Fig. 1D and F). We identified 11 cell subpopulations: CD4 T cells, Tregs, B cells, CD8 T cells, tumor cells, plasma cells, monocytes, fibroblasts, endothelial cells, mast cells, and smooth muscle cells (Fig. 1B). Mapping cells from left- and right-sided colorectal cancer tissues onto UMAP plots revealed distinct distribution differences for plasma cells, Tregs, and tumor cells (Fig. 1C).

Comparative analysis of cell proportions between the two groups showed a higher proportion of tumor cells and plasma cells in right-sided colorectal cancer, while Tregs, monocytes, and mast cells were more abundant in left-sided colorectal cancer (Fig. 1E). We further highlighted the top 5 genes for each cell subpopulation: Tregs (IL7R, FYB1, SARAF, SLC2A3, FXYD5), fibroblasts (COL1A1, COL1A2, COL3A1, IGFBP5, CXCL14), tumor cells (KRT8, PHGR1, KRT18, TFF3, ELF3), and CD8 T cells (GZMB, GZMA, CCL4, NKG7, CCL5) (sFig. 1C). Differential gene expression analysis of all cells revealed various degrees of up- and down-regulation, which may be related to the different functions of each cell type (Fig. 1G).

### Tumor cells in right-sided colorectal cancer exhibit higher circadian-rhythm disruption scores than those in left-sided colorectal cancer

**Fig. 2:**
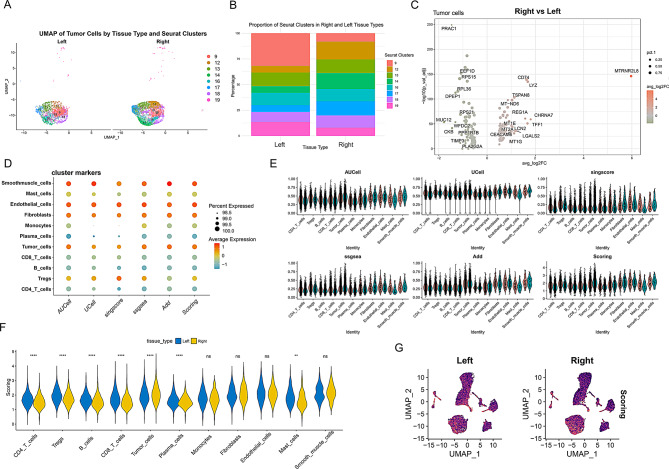
Higher expression levels of circadian rhythm genes in tumor cells of right-sided colorectal cancer. (**A**) UMAP plot showing the distribution of tumor cells from left-sided and right-sided colorectal cancer. Each color represents a specific cell subpopulation (Seurat clusters). The x-axis and y-axis represent the two main UMAP components. (**B**) Bar graph depicting the proportions of cell subpopulations in tumor cells from left-sided and right-sided colorectal cancer. Different colors represent different cell subpopulations. (**C**) Volcano plot illustrating differential gene expression in tumor cells between right-sided and left-sided tissues. The x-axis shows the fold change in gene expression (avg_log2FC), and the y-axis represents the significance of gene expression changes (-log10 p-value). (**D**) Dot plot showing circadian rhythm phenotype scores across different cell populations. The size of the dots represents the percentage of gene set expression, while the color intensity indicates the average expression level of the gene set. (**E**) Violin plot displaying the scoring of different cell populations using five different scoring methods (AUCell, UCell, singscore, ssGSEA, AddModuleScore). Red represents left-sided colorectal cancer, and cyan represents right-sided colorectal cancer. (**F**) Violin plot illustrating the distribution of scores in different cell populations from left-sided and right-sided tissues. Blue represents left-sided tissue, and yellow represents right-sided tissue. Statistical significance levels are indicated as follows: **** *p* < 0.0001, ** *p* < 0.01, ns indicates no significant difference. (**G**) UMAP plot showing the distribution of scores across different cell populations in left-sided and right-sided tissues. The color of each dot represents the score level

We separately presented the dimensionality reduction clustering plots for tumor cells and found differences between left-sided and right-sided colorectal cancer cells. The distribution of left-sided colorectal cancer cells was relatively uniform, with cell subpopulations 9 and 19 being more concentrated. In contrast, right-sided colorectal cancer cells, particularly subpopulations 14, 16, 17, and 12, were more concentrated (Fig. 2A). Cell-proportion analysis indicated that subpopulation 9 was most abundant in left-sided CRC, whereas subpopulation 12 predominated in right-sided CRC; conversely, subpopulation 9 was the least abundant in right-sided CRC (Fig. 2B). Further comparison of gene expression differences between right-sided and left-sided colorectal cancer tumor cells revealed that genes such as MTRNR2L8, CHRNA7, TFF1, and LYZ were more highly expressed in right-sided colorectal cancer (Fig. 2C). We utilized five phenotypic scoring methods, including AUCell, UCell, singscore, ssgsea, and Add, to determine the extent of circadian rhythm disruption scores in various cells. The analysis revealed that endothelial cells, fibroblasts, and tumor cells exhibited relatively higher circadian rhythm disruption scores (Fig. 2D). Further normalization and statistical evaluation across five scoring algorithms demonstrated that tumor cells from right-sided colorectal cancer displayed significantly higher circadian rhythm disruption scores than those from left-sided tumors (Fig. 2E-G).

### HCR TC exhibits stronger communication links with fibroblasts compared to LCR TC

**Fig. 3:**
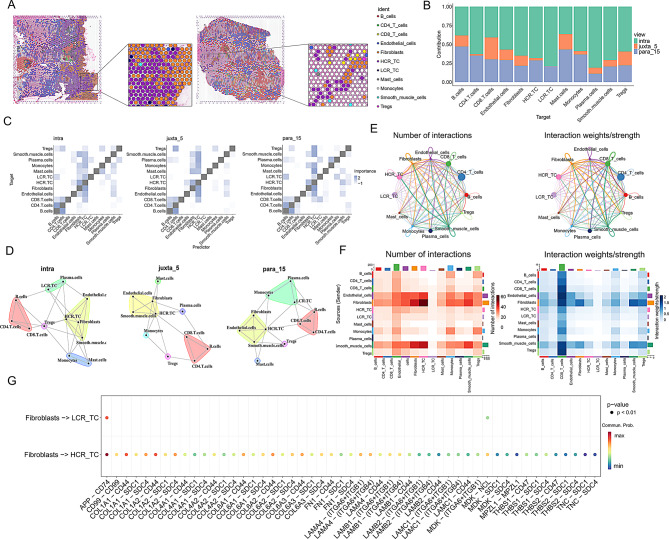
Spatial Distribution and Cell Communication Relationships between HCR Tumor Cells and LCR Tumor Cells. (**A**) Spatial deconvolution analysis of cell distribution in right-sided colorectal cancer. (**B**) Grouped stacked bar chart showing the contribution levels of different cells at varying distances. (**C**) Heatmap depicting cell correlations at different distances. (**D**) Cell interaction network diagram at different distances. (**E**, **F**) The network graph and heatmap display the number and strength of communication links between different cell types. (**G**) Dot plot of receptor–ligand pairs linking fibroblasts with HCR TC or LCR TC, where color intensity indicates mean expression

We mapped single-cell transcriptomic profiles onto spatial sections via deconvolution and found HCR TC detected in a greater number of spots, indicating broader spatial prevalence. Moreover, there were more fibroblasts distributed around the HCR TC spots (Fig. 3A). Figure 3B shows that most cells primarily exert their influence within the intra category. In spatial interaction analysis, we observed that HCR TC and fibroblasts have tighter interactions across various ranges, further suggesting a potential association between HCR TC and fibroblasts (Fig. 3C and D). Based on CellChat analysis of single-cell data, we observed that HCR TC exhibited stronger communication interactions with fibroblasts, endothelial cells, and smooth muscle cells (Fig. 3E and F). Figure 3G illustrates that the communication received by HCR TC from fibroblasts was significantly stronger than that received by LCR TC. Additionally, in terms of ligand-receptor interactions, the communication involving HCR TC was far more abundant than that involving LCR TC.

### Expression characteristics of circadian rhythm differential genes between groups analyzed by single-cell pseudotime and spatial transcriptomics

**Fig. 4:**
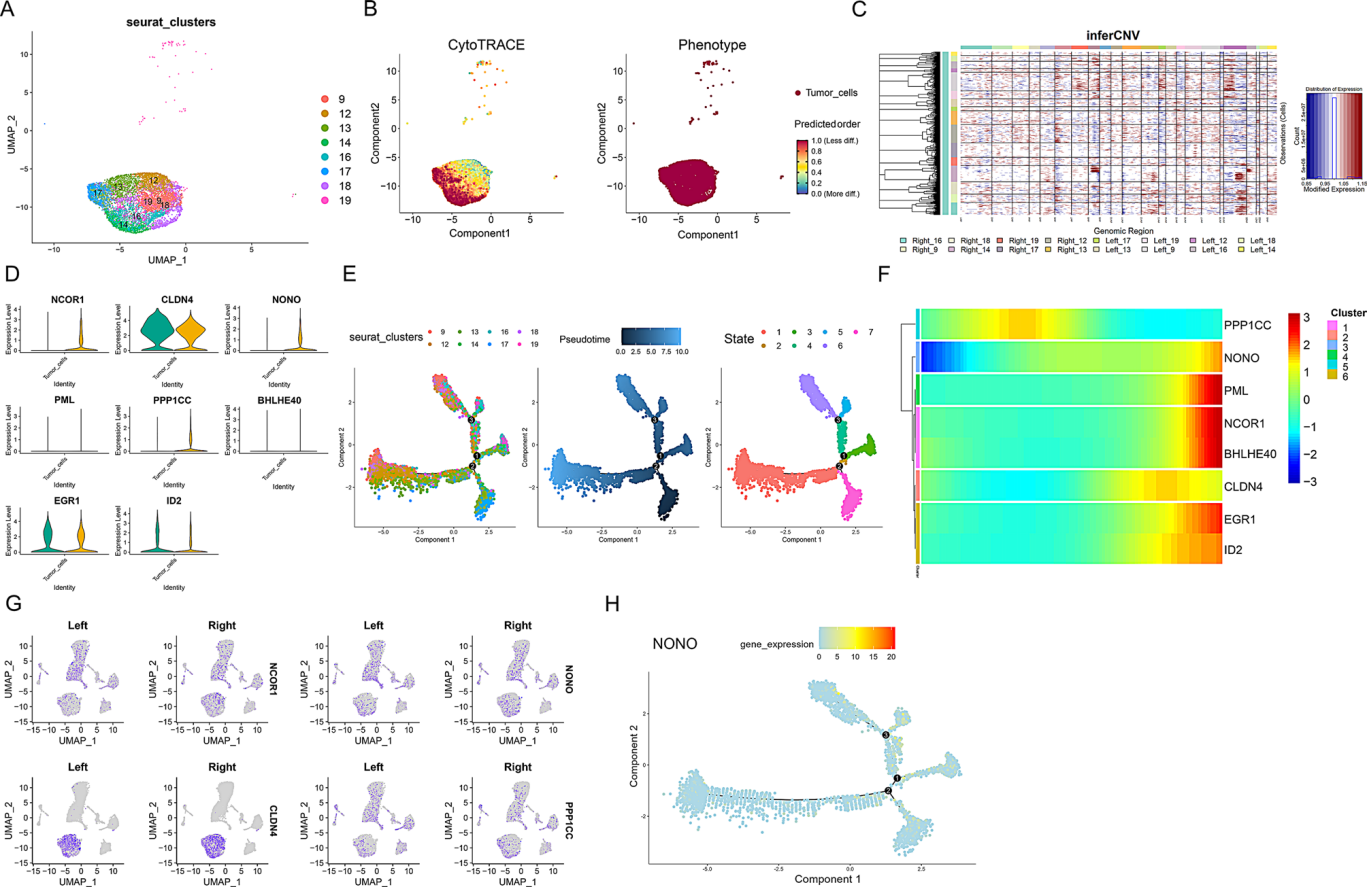
Pseudotime analysis reveals the expression of differential circadian rhythm genes in right-sided colorectal cancer. (**A**) UMAP plot showing the distribution of clustered tumor cells. Each dot represents a cell, and different colors indicate different Seurat clusters, reflecting the clustering of cell populations. (**B**) CytoTRACE and Phenotype analysis. The left panel shows the CytoTRACE predicted cell developmental stages, with colors ranging from red (less differentiated) to green (more differentiated); the right panel illustrates the phenotype distribution of tumor cells, with red dots representing tumor cells. (**C**) inferCNV heatmap displaying copy number variations across genomic regions. Red indicates amplifications and blue indicates deletions, helping to identify CNV characteristics in tumor cells. (**D**) Violin plots showing differential expression levels of circadian rhythm genes in tumor cells from left-sided and right-sided colorectal cancer. Each violin plot represents a gene, with green representing left-sided and yellow representing right-sided colorectal cancer. (**E**) Single-cell trajectory analysis. The left panel shows the distribution of cells from different Seurat clusters along the trajectory, the middle panel shows the pseudotime trajectory of cells, and the right panel displays the distribution of cell states, with different colors representing different states. (**F**) Heatmap showing the expression of differential genes across different cell states. Colors range from blue (low expression) to red (high expression), helping to identify gene expression patterns across cell states. (**G**) UMAP plots showing the expression distribution of specific genes in left-sided and right-sided tissues. Each plot illustrates the expression of a gene in both tissues. (**H**) Single-cell trajectory analysis showing the expression distribution of the NONO gene along the cell trajectory. Colors range from blue (low expression) to red (high expression)

**Fig. 5:**
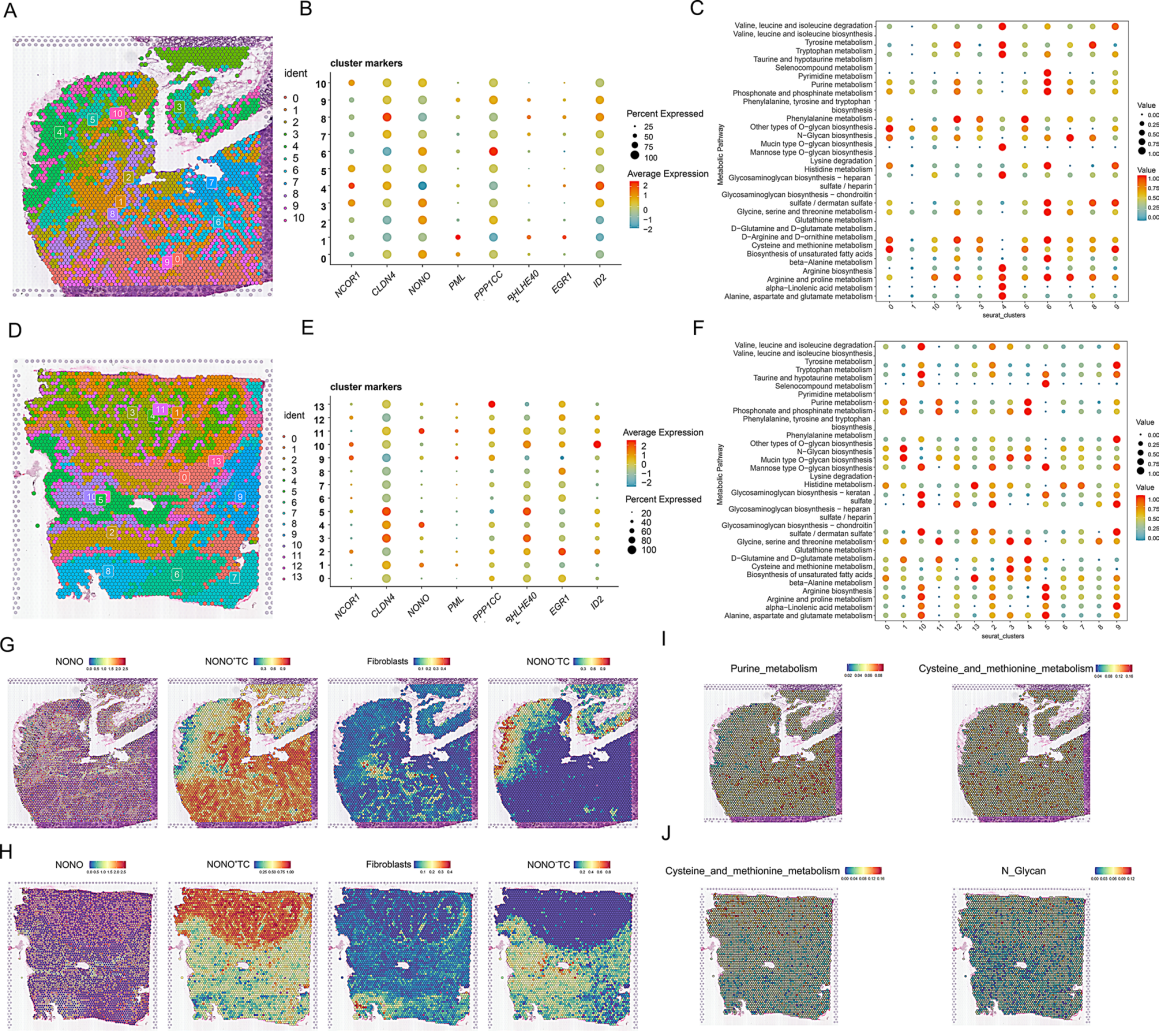
Spatial transcriptomics reveal the spatial proximity between NONO ^+^ tumor cells and fibroblasts, which may be associated with specific metabolic functions. (**A**, **D**) Spatial clustering maps of colorectal cancer tissue sections. Different colors represent distinct cell subpopulations identified through spatial transcriptomics, illustrating the spatial organization of various cell types within the tissue. (**B**, **E**) Dot plots showing the expression of differential circadian rhythm genes between two groups across various subpopulations. The size of the dots represents the percentage of cells expressing the marker gene, and the color intensity indicates the average expression level. (**C**, **F**) Dot plots displaying metabolic pathway enrichment scores across different cell subpopulations. Each dot represents a pathway, with the size of the dots indicating the significance of enrichment, and the color intensity representing the average expression level of the pathway. (**G**, **H**) Spatial distribution maps showing the gene NONO, NONO⁺ TC, NONO- TC, and fibroblasts within tissue sections. Color gradients indicate the levels of gene expression or cell distribution. (**I**, **J**) Spatial enrichment maps of specific metabolic pathways. Color gradients represent the activity levels of the pathways

To further investigate the reasons for phenotypic differences between the left and right sides, we analyzed the differential genes associated with the phenotype between the two groups. We found that NCOR1, NONO, and PPP1CC were more highly expressed in right-sided colorectal cancer, while CLDN4, EGR1, and ID2 were more highly expressed in left-sided colorectal cancer (Fig. 4D). Complementing these observations, correlation analysis between CRD scores and NONO expression at the single-cell level (Table S6) revealed a statistically robust positive association (Pearson *r* = 0.262; Spearman ρ = 0.348; both *p* < 0.001). Additionally, CRD scores were displayed separately for NONO⁺ TC and NONO^–^TC, revealing consistently higher scores in NONO⁺ TC than in NONO^–^TC in both left-sided and right-sided CRC (sFig. 8). UMAP plots confirmed similar expression patterns for these genes across sides (Fig. 4G). CytoTRACE analysis was used to explore the starting points of cell differentiation, revealing that tumor cell clusters 17 and 14 might be the initiation points of tumor cell differentiation (Fig. 4A and B). Consistent with the CytoTRACE findings, InferCNV analysis showed that tumor cell cluster 12 had more extensive copy number variations, suggesting a higher level of genomic instability (Fig. 4C). Based on these analyses, we identified the starting points of cell differentiation and different differentiation states (Fig. 4E). Consequently, we inferred that the expression of NONO, NCOR1, BHLHE40, and EGR1 increases along pseudotime, whereas PPP1CC and CLDN4 are preferentially expressed at other developmental stages (Fig. 4F and H). We further selected spatial transcriptomics data from four patients for quality control and dimensionality reduction clustering (sFigs. 2 and Fig. 5A and D). Combining pathological staining of colorectal cancer tissue samples and clustering results from the slices, we found that NONO gene expression was predominantly located in tumor nests (Fig. 5B and E). Similarly, NONO⁺ TC were also more frequently found in tumor nests. To visualize their spatial relationship, we mapped the distribution of fibroblasts alongside the abundance scores of NONO^+^ and NONO^−^ TC. These plots reveal a clear spatial co-localization between regions of high fibroblast density and areas with a high abundance of NONO⁺ TC, a pattern not observed for NONO^−^TC (Fig. 5G and H). We also examined the spatial distribution of certain metabolic characteristics and found metabolic activities such as purine metabolism, cysteine and methionine metabolism, and N-glycan biosynthesis, indicating that NONO may be associated with these specific tumor metabolic activities (Fig. 5C, F, I, and J).

### Specific interaction between NONO⁺ TC and fibroblasts

**Fig. 6:**
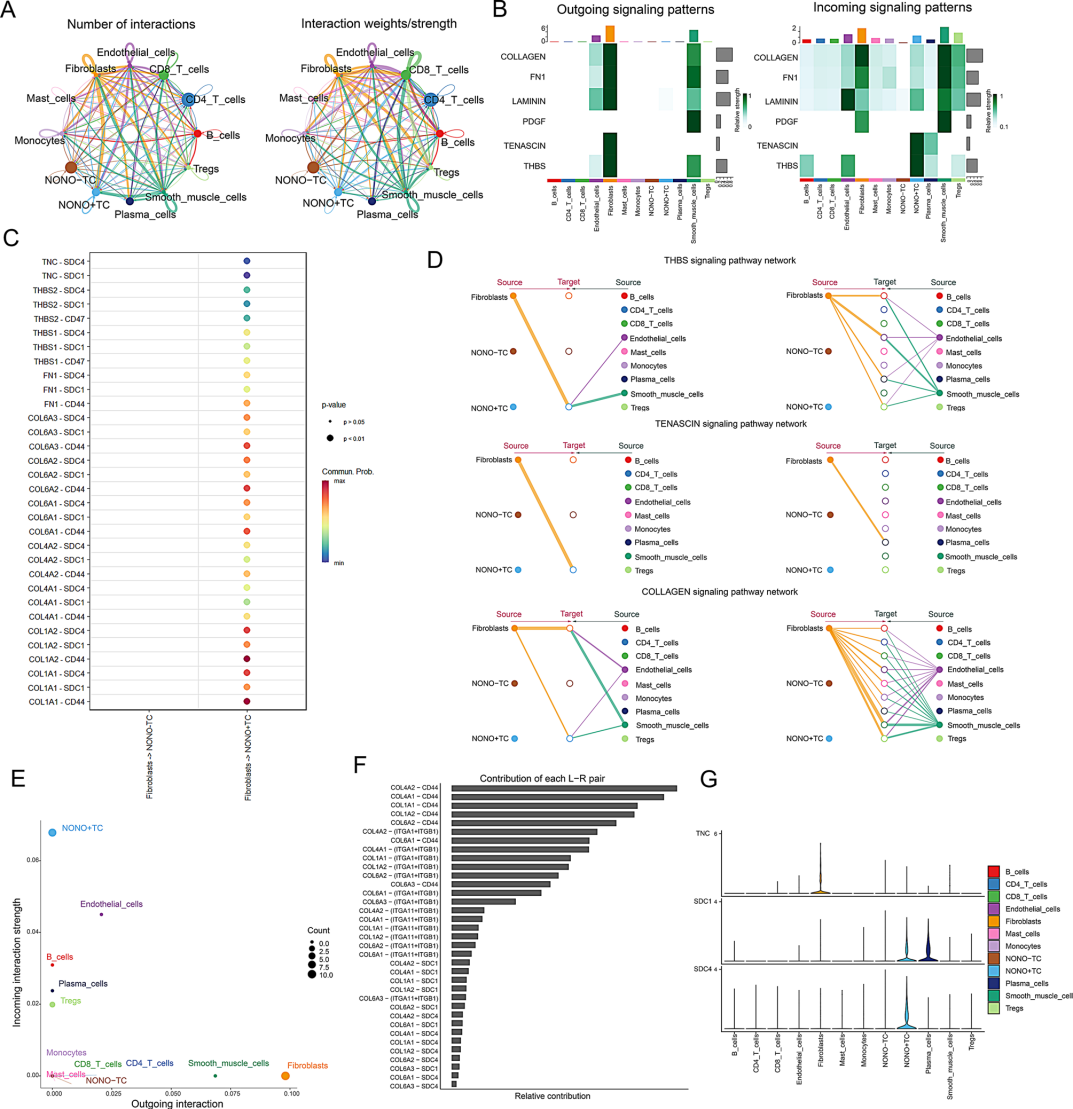
Interactions between NONO ^+^ TC and fibroblasts in right-sided colorectal cancer. (**A**) The left panel shows the number of interactions between different cell types, and the right panel shows the weight strength of these interactions. Nodes represent different cell types, with node size proportional to the number of interactions and edge thickness representing interaction strength. (**B**) The left panel shows the outgoing signaling patterns of cells, and the right panel shows the incoming signaling patterns. The heatmap color indicates the strength of signal transduction, with darker colors representing stronger signals. The vertical axis lists signaling pathways, and the horizontal axis lists cell types. (**C**) Dot plot showing the interaction strength between ligands and receptors. The color intensity represents the expression level of the receptors for each ligand. (**D**) Network diagrams for three signaling pathways (THBS, COLLAGEN), showing intercellular interactions. Nodes represent cell types, edges represent signaling relationships between cells, and edge thickness represents signal strength. (**E**) Scatter plot showing the signaling strength of different cell types in sending or receiving signals. The horizontal axis represents outgoing signal strength, the vertical axis represents incoming signal strength, and dot size represents the number of interactions. (**F**) Bar graph showing the relative contribution of different ligand-receptor pairs to overall signal transduction. The length of the bars represents the magnitude of the relative contribution. (**G**) Violin plots showing the expression distribution of SDC1 and SDC4 across different cell types. The horizontal axis represents cell types, the vertical axis represents expression levels, and different colors represent different cell types

Given the critical role of NONO in circadian rhythms and the potential colocalization of NONO⁺ TC with fibroblasts, we further investigated the communication activities between NONO⁺ TC and other cells based on single-cell data (Fig. 6A). From the perspective of signal output, communication between fibroblasts and NONO⁺ TC was significantly higher compared to NONO^−^ TC (sFig. 3A). NONO⁺ TC also appeared to be more active than NONO^−^ TC (sFig. 3B and C). This study focused on key pathways of communication between NONO⁺ TC and fibroblasts, including COLLAGEN, FN1, LAMININ, PDGF, TENASCIN, and THBS. It was found that except for PDGF, fibroblasts were the main source of the other signals, while NONO⁺ TC primarily received these signals. Notably, TENASCIN and THBS signals were predominantly emitted by fibroblasts and not involved in self-communication, with NONO⁺ TC being the main recipients of these signals. This indicates that TENASCIN and THBS play crucial roles in the communication between fibroblasts and NONO⁺ TC (Fig. 6B and E). Hierarchical diagrams further illustrated the specific communication patterns of TENASCIN, THBS, and COLLAGEN, revealing that TENASCIN was exclusively present between NONO⁺ TC and fibroblasts. This suggests a unique cell communication pattern between NONO⁺ TC and fibroblasts (Fig. 6D). Additionally, we highlighted the expression and relationship of ligand-receptor pairs involved in TENASCIN and THBS communication. High-expression pairs such as SDC4, COL1A1, SDC1, and CD44 may play key roles in mediating communication between the two cell types (Fig. 6C and F-G). To further resolve cancer-associated fibroblast heterogeneity, we performed a second round of dimensionality-reduction clustering on fibroblasts, delineating three CAF states—inflammatory Cancer-Associated Fibroblasts(iCAF), myoepithelial-like Cancer-Associated Fibroblasts(myCAF) and antigen-presenting Cancer-Associated Fibroblasts(apCAF) (sFig.4A). Cell–cell communication analysis showed that NONO⁺ TC interact far more strongly with the myCAF subset than with the other CAF phenotypes (sFig. 4B–C). Network role profiling confirmed that myCAFs lie in the upper-right quadrant of the signaling-role scatter plot, displaying the highest combined outgoing and incoming strengths of all cell types, whereas NONO⁺ TC rely chiefly on CAF-derived inputs; this selective crosstalk is likely to dictate their spatial distribution and phenotypic state (sFig. 4D).

### Single-cell spatial deconvolution reveals spatial interactions of NONO⁺ TC

**Fig. 7:**
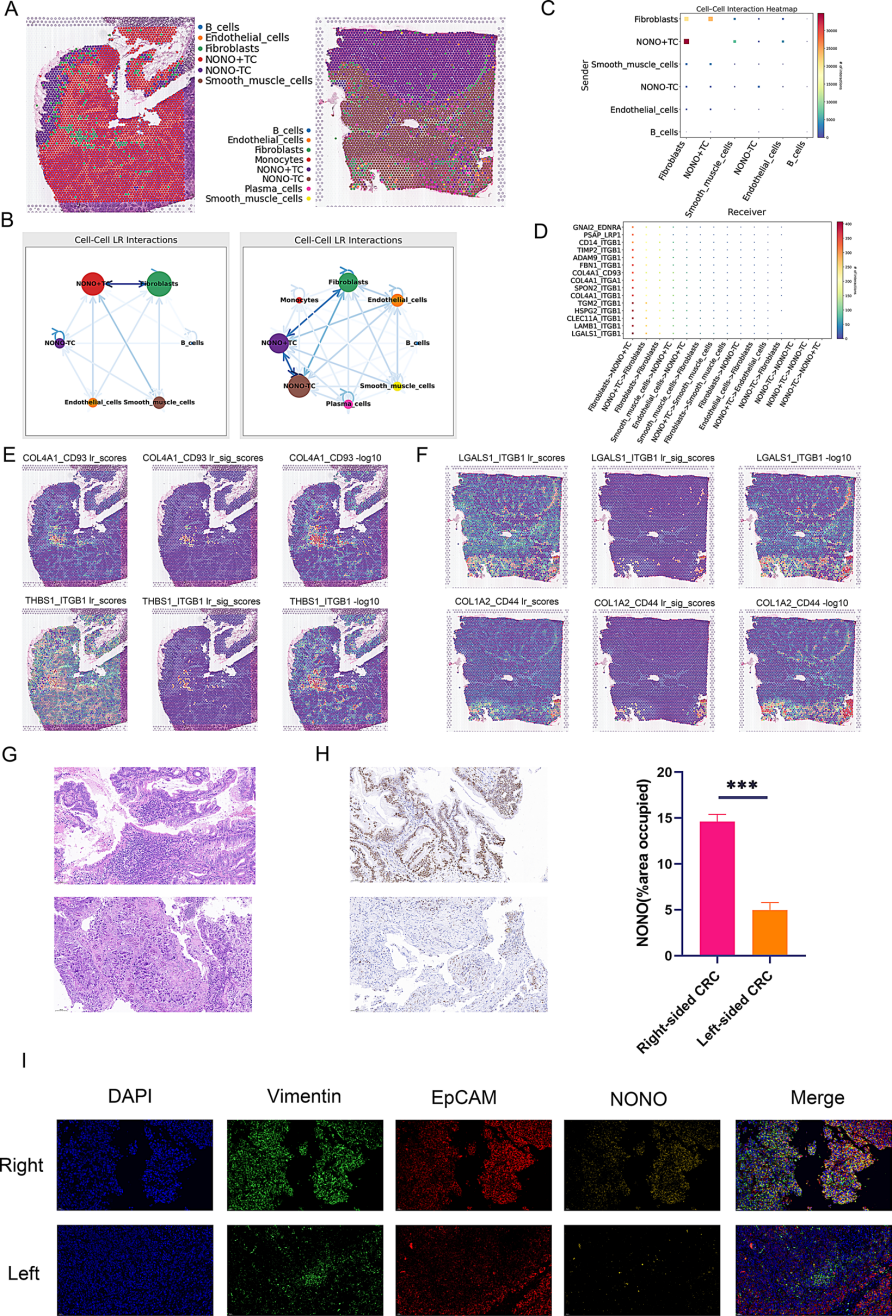
Spatial Deconvolution and Multiplex Fluorescence Reveal Spatial Characteristics of Interaction between NONO ^+^ TC and Fibroblasts. (**A**) Spatial mapping of single-cell–annotated cell types onto the tissue section. Each dot represents a spatial spot, and colors denote distinct cell types. (**B**) Cell–cell communication network illustrating intercellular interactions; darker edge colors indicate stronger communication strength. (**C) ** Cell-cell communication heatmap showing interaction strength between cell types;darker colors indicate stronger communication. (**D**) Dot plot showing the expression of key ligand-receptor pairs involved in cell interactions. The color intensity indicates the expression level of the key ligands and receptors. (**E**-**F**) Spatial distribution of key ligand-receptor interactions between NONO⁺ TC and fibroblasts. These panels illustrate the specific spatial locations of the interactions. (**G**) HE staining results of left-sided and right-sided colorectal cancer. The staining highlights the histological differences between the two sides. (**H**) Immunohistochemical staining and quantitative analysis of NONO in left-sided and right-sided colorectal cancer. The left panel shows representative images, and the right panel presents the quantification of NONO expression levels. (**I**) Multiplex immunofluorescence staining of EpCAM, Vimentin, and NONO in left-sided and right-sided colorectal cancer. The left panel shows representative images, and the right panel presents the quantification of staining intensity for these markers

We further performed spatial deconvolution on right-sided colorectal cancer data to analyze which cell types are most likely to be present at each spot in the tissue slices and their potential interactions. Following cell type deconvolution with RCTD, we generated a spatial map assigning a dominant cell identity to each spot. This analysis confirmed that NONO⁺ TC predominantly form distinct tumor nests. Notably, at the boundaries of these NONO⁺ TC clusters, we consistently observed an enrichment of annotated fibroblasts, creating an interwoven pattern indicative of a direct cellular interface (Fig. 7A). Spatial cell communication analysis also indicated that fibroblasts have more prominent interactions with NONO⁺ TC compared to NONO^−^ TC (Fig. 7B and C). We further illustrated the spatial distribution of certain ligand-receptor pairs, such as COL4A1-CD93, THBS1-ITGB1, LGALS1-ITGB1, and COL1A2-CD44, which play crucial communication roles between fibroblasts and NONO⁺ TC. These ligand–receptor patterns were consistent with the results from CellChat analysis (Fig. 7D-F). Based on tissue samples obtained from clinical patients, we validated the bioinformatics analysis experimentally. Figure 7G shows HE staining of left-sided and right-sided colorectal cancer patients, clearly displaying tumor distribution within the tissues. Immunohistochemistry studies of both sides revealed that NONO is highly expressed in right-sided colorectal cancer, with significantly more NONO-expressing cells than in the left side (Fig. 7H). Multiplex immunofluorescence experiments demonstrated the colocalization relationship between NONO⁺ TC and fibroblasts, with a higher distribution of NONO⁺ TC in the right side compared to the left side (Fig. 7I). This suggests a distinctive role of NONO in right-sided colorectal cancer, different from that in left-sided colorectal cancer.

We next quantified the spatial-scale associations between NONO⁺ TC and all other cell types (Table S1). Fibroblasts emerged as the dominant predictor of NONO⁺ TC abundance at both the intra- (imp = 55.21) and juxta-layer (imp = 40.04) views, far exceeding the importance of any other cell type (Table S3). At the para layer fibroblasts still contributed significantly (imp = 25.06, *p* < 0.001), and—reciprocally—NONO⁺ TC ranked among the top predictors of fibroblast abundance in the fibroblast model (imp = 10.42), indicating a bidirectional spatial coupling (Table S2).Model performance metrics corroborated these findings: the intra-view model for NONO⁺ TC achieved R² = 0.9018, which increased to 0.9115 after incorporating juxta- and para-level covariates, demonstrating additional explanatory power from non-autonomous spatial structure. For the fibroblast model, R² rose from 0.8106 to 0.8228 upon multi-layer integration (Table S4), showing that spatial features account for the majority of fibroblast variability and that multiscale analysis further improves model fit. Collectively, these results provide robust statistical support for the imaging-based co-localisation and suggest that fibroblasts, through multiscale spatial distributions, critically influence the organisation of NONO⁺ TC within the colorectal tumor microenvironment.

### NONO is upregulated in colorectal cancer and promotes malignant phenotypes by modulating circadian rhythm and fibroblast-related signaling pathways

**Fig. 8:**
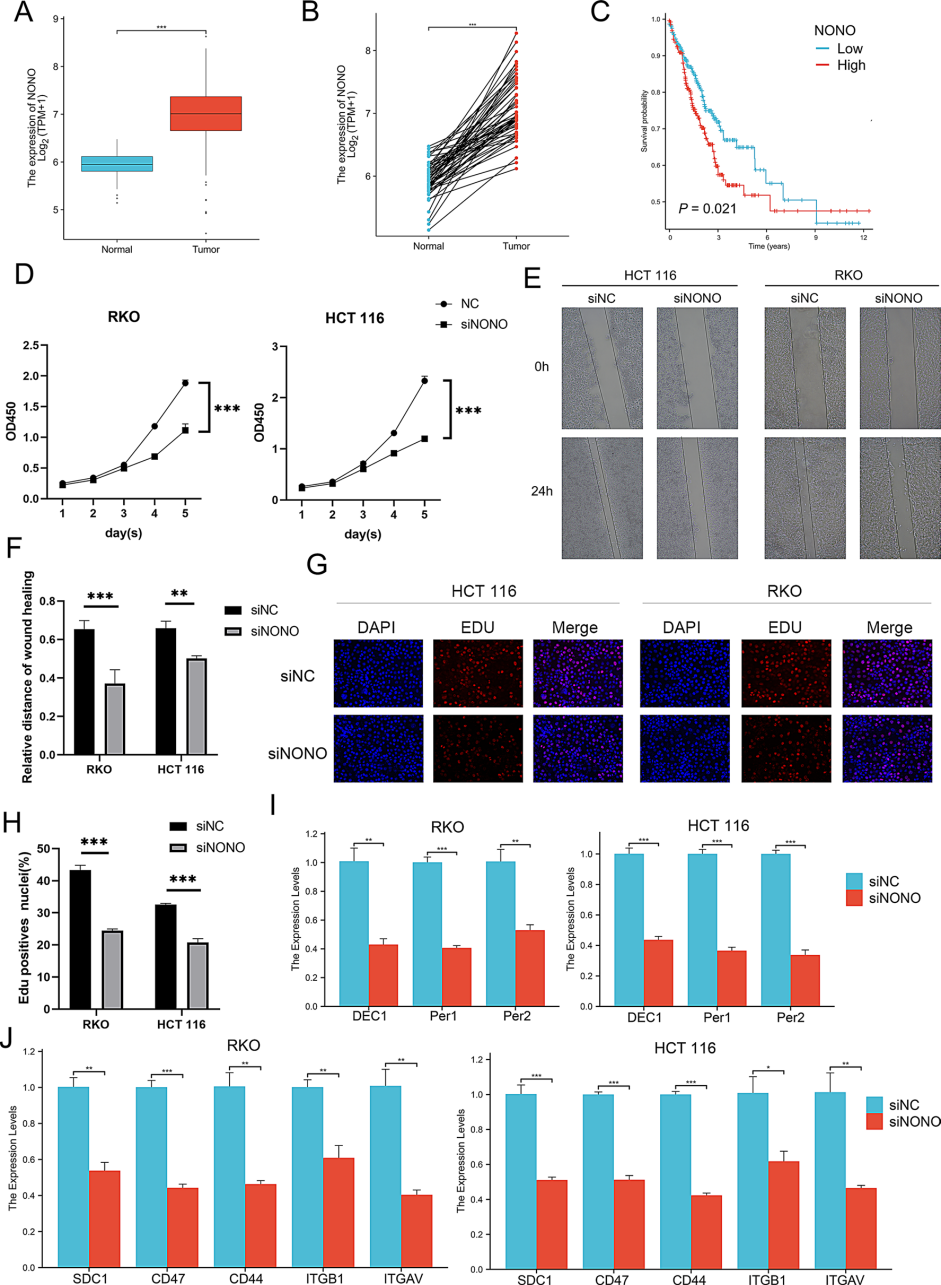
Expression of NONO in colorectal cancer and its role in regulating malignant phenotypes. (**A**-**B**) Comparison of NONO expression between colorectal cancer (CRC) tissues and adjacent normal tissues in the TCGA database. Differential expression is shown for (**A**) paired and (**B**) unpaired samples, revealing significant upregulation of NONO in tumor tissues. (**C**) Kaplan-Meier survival analysis for the TCGA cohort, with patients stratified by high and low NONO expression levels. (**D**) Cell proliferation of RKO and HCT-116 cells at different time points after NONO knockdown, as measured by CCK-8 assay. (**E**-**F**) Assessment of cell migration ability upon NONO knockdown using a wound healing assay. (**E**) Representative images of the scratch wound at 0 and 24 h. (**F**) Quantitative analysis of the wound closure rate. (**G**-**H**) Further validation of cell proliferation after NONO knockdown using an EdU incorporation assay. (**G**) Representative fluorescence images of EdU (proliferating cells) and Hoechst (all nuclei). (**H**) Quantification of the EdU-positive rate. (**I**) Relative mRNA expression of key circadian rhythm genes following NONO knockdown, as measured by qPCR. (**J**) Relative mRNA expression of key receptors on tumor cells, which are responsible for receiving fibroblast-derived signals, following NONO knockdown, as measured by qPCR

To investigate the clinical relevance and biological function of NONO in CRC, we first analyzed the TCGA database, finding that the mRNA expression level of NONO was significantly higher in CRC tumor tissues than in adjacent normal tissues (Fig. 8A-B), and that its high expression was closely associated with poorer patient prognosis (Fig. 8C). To validate function, we performed siRNA-mediated NONO knockdown in RKO and HCT116 cells. Functional analyses showed that silencing NONO significantly inhibited the proliferation and migration of CRC cells, a finding consistently verified by CCK-8, wound healing, and EdU incorporation assays (Fig. 8D-H). To further elucidate its molecular mechanism, we assessed downstream gene expression changes via qPCR. NONO knockdown significantly downregulated the core clock gene PER2 and the oncogene DEC1 (Fig. 8I) and markedly reduced tumor-cell expression of key fibroblast-signal receptors, including ITGB1, SDC1, and CD47 (Fig. 8J). Collectively, these findings establish NONO as a key pro-tumorigenic factor in CRC, whose function is linked not only to regulating the intrinsic circadian machinery but also to enhancing the tumor cell’s receptiveness to pro-tumorigenic signals from the microenvironment.

**Fig. 9:**
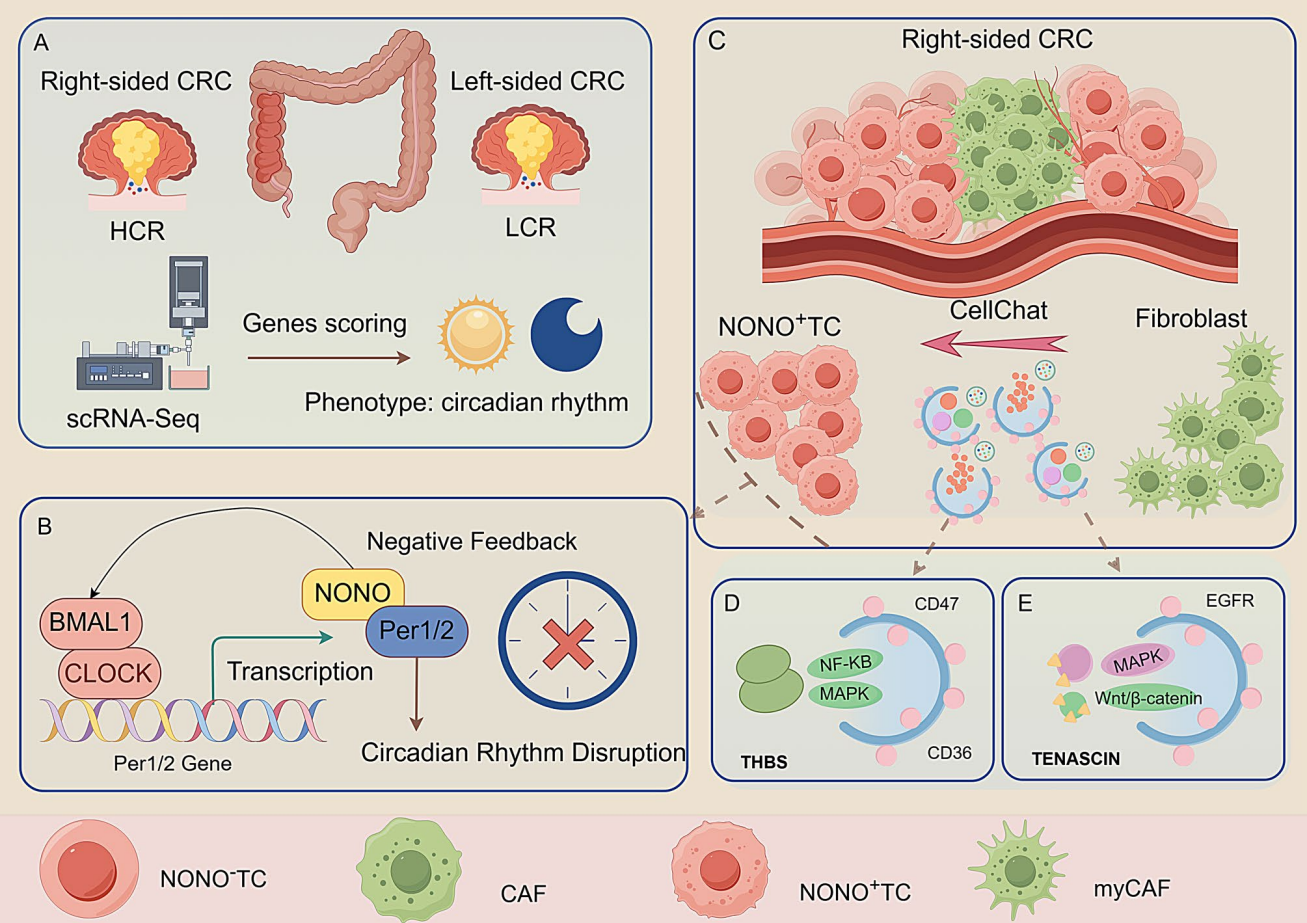
A schematic model illustrating the NONO-centric mechanism linking circadian rhythm disruption to the malignant microenvironment in right-sided colorectal cancer. This schematic depicts a circadian-regulated, NONO-centered mechanism of tumor–stroma interaction in right-sided colorectal cancer. Integrating single-cell and spatial transcriptomics, we show that tumor cells in right-sided disease exhibit markedly higher circadian rhythm–disruption scores, with positive expression of the core clock factor NONO emerging as a defining molecular feature of this phenotype. Mechanistically, NONO amplifies circadian dysregulation by modulating downstream core clock genes and cell-cycle inhibitors. Within the tumor microenvironment, this NONO-driven rhythmic disturbance reshapes intercellular communication, establishing a selective exchange between NONO⁺ TC and fibroblasts. Further analysis indicates that this crosstalk is mediated primarily by TENASCIN and THBS signaling pathways, in which fibroblasts act as the dominant signal senders while NONO⁺ TC are remodeled into highly efficient signal receivers, thereby fostering the emergence of a malignant niche

### Construction of a colorectal cancer prognostic model based on circadian rhythm-related genes

We constructed a four-gene, circadian rhythm–related prognostic model for colorectal cancer using LASSO regression. sFig 6A shows the LASSO coefficient path. sFig 6B displays the cross-validation curve, with the minimum-λ model selected as optimal. According to the forest plot, NOS2 and PPARGC1A are associated with reduced risk, whereas DRD4 and NGFR are linked to increased risk (sFig. 6C). Based on this four-gene prognostic model, patients were stratified into high- and low-risk groups, and survival analysis indicated that patients in the high-risk group had significantly poorer outcomes (*p* < 0.0001) (sFig. 6D). The time-dependent ROC curves assessed the model’s predictive accuracy at 1, 2, and 5 years, with AUC values ranging from 0.703 to 0.716, demonstrating good model precision (sFig. 6E). To further validate the generalizability of our model, the GSE39582 dataset, complete with clinical information, was sourced from the Gene Expression Omnibus (GEO) database to serve as an external validation cohort. We first performed batch effect correction on the integrated data, and PCA confirmed that the batch effects were effectively eliminated (sFig. 5). In the validation cohort, survival analysis demonstrated that the model could significantly stratify colorectal cancer patients into high- and low-risk prognostic groups (sFig. 6F). However, the time-dependent ROC analysis revealed suboptimal predictive accuracy in the validation cohort, indicating that the model’s predictive performance requires further optimization and refinement (sFig. 6G).

## Discussion

The role of circadian rhythms in human health is critically important. Circadian rhythms are intrinsic time-keeping mechanisms that regulate various physiological processes over a 24-hour cycle. These rhythms are controlled by core clock genes, which maintain homeostasis in the body through positive and negative feedback loops. Disruption of circadian rhythms can lead to significant health problems, including an increased risk of cancer and the promotion of cancer progression [21]. In the immune system, circadian rhythms influence the body’s response to antigens and inflammation by regulating the balance of cytokines produced by T-helper cells. Disruption of the sleep-wake cycle, an essential component of circadian rhythms, can impair immune function and promote chronic inflammation, which is a known risk factor for cancer. Additionally, circadian rhythms affect cell cycle regulation, apoptosis, DNA repair, and metabolic processes, all of which are crucial for tumorigenesis and cancer development. Mutations or dysfunctions in core clock genes can promote tumorigenesis by affecting these cellular functions. For instance, studies have shown that mutations in CLOCK and BMAL1 can either enhance or suppress tumor growth, depending on the specific context and tissue type [22].

Beyond its role as a clock-associated protein, NONO is a multifunctional RNA-binding protein whose key post-transcriptional function—alternative splicing—may serve as a critical bridge linking circadian regulation to microenvironmental crosstalk. Studies have shown that NONO can regulate the alternative splicing of the tumor suppressor PTEN pre-mRNA, where elevated NONO expression promotes the production of a functionally impaired PTEN isoform, leading to sustained activation of the PI3K/AKT signaling pathway and, consequently, enhanced tumor proliferation and invasion [23]. Activation of the PI3K/AKT pathway also augments the ability of tumor cells to secrete cytokines and growth factors, thereby further stimulating fibroblasts. In glioblastoma, NONO cooperates with PSPC1 to bind GPX1 pre-mRNA, regulating its alternative splicing and thereby influencing tumor growth, invasion, and redox homeostasis [24]. Redox imbalance is a hallmark of the tumor microenvironment that profoundly affects the function of stromal cells such as fibroblasts. Under hypoxic conditions, NONO is upregulated as a hypoxia-inducible gene and directly binds to and stabilizes HIF-1α mRNA, thereby amplifying hypoxia signaling to promote tumor progression and angiogenesis [25]. This directly links NONO function to hypoxia, one of the key drivers shaping the malignant microenvironment. In summary, as a member of the circadian rhythm network, aberrant expression or dysregulation of NONO in cancer cells can, through its RNA-binding protein functions, post-transcriptionally regulate a series of key genes involved in proliferation, invasion, metabolism, and extracellular matrix remodeling. This endows tumor cells with an enhanced capacity for bidirectional communication with stromal cells such as fibroblasts, ultimately fostering the establishment of a malignant tumor microenvironment.

In our study, right-sided CRC exhibited significantly higher circadian-rhythm disruption (CRD) scores than left-sided CRC, suggesting location-specific biological effects of circadian regulation. The differences in genetic and molecular characteristics between right-sided and left-sided colorectal cancers may be important reasons for the differences in circadian rhythm gene expression. Studies have shown that right-sided colorectal cancer usually has higher microsatellite instability (MSI), whereas left-sided colorectal cancer more commonly exhibits chromosomal instability (CIN). MSI and CIN represent two different patterns of genomic instability that may influence the expression and regulation of circadian rhythm genes through different mechanisms. Differences in gut-microbiota composition may also contribute substantially to side-specific variation in circadian-gene expression. Previous studies have found that the microbial diversity and composition in right-sided colorectal cancer differ significantly from those in left-sided colorectal cancer. The gut microbiota can influence the host’s circadian rhythms through various mechanisms, such as producing circadian rhythm-regulating metabolites and affecting the host’s immune and endocrine functions. We also found a higher interaction between tumor cells with higher circadian rhythm disruption scores and fibroblasts. Additionally, certain metabolic features, such as purine metabolism, cysteine and methionine metabolism, and N-glycan metabolism, may play a key role. These metabolic pathways not only regulate the energy requirements of cells but may also play important roles in the growth and survival of tumor cells. Changes in N-glycan metabolism in tumor cells and fibroblasts are closely related to cancer progression and cell migration. N-glycans are essential components of glycoproteins and play critical roles in cell surface molecular recognition and signal transduction. The increased branching and modification of N-glycans in tumor cells promote the retention of growth factor receptors on the cell surface, thereby enhancing cell proliferation and migration [26]. Meanwhile, the regulation of N-glycan metabolism in fibroblasts affects the formation of the extracellular matrix and cell migration, especially in cancer-associated fibroblasts, which is closely related to the establishment of the tumor microenvironment [27]. This dual role suggests that N-glycan metabolism promotes both tumor-cell invasiveness and survival, as well as CAF-mediated microenvironment formation and maintenance. This further drives progression and metastasis. The interaction with CAFs, which secrete potent growth factors, places high proliferative demands on tumor cells. The observed hyperactivity of purine metabolism in NONO⁺ TC likely reflects a metabolic adaptation to meet this demand, supplying the essential purine nucleotides for DNA and RNA synthesis required for rapid cell division [28]. Moreover, the intense crosstalk between tumor cells and CAFs is known to create a microenvironment with high levels of reactive oxygen species (ROS). The upregulation of cysteine and methionine metabolism is therefore a crucial survival strategy for NONO⁺ TC, as it provides the cysteine necessary for synthesizing glutathione (GSH), the primary antioxidant that detoxifies ROS and enables the cells to thrive in this CAF-induced hostile environment [29].

We conducted differential expression analysis of circadian rhythm genes in tumor cells from both sides and found that NONO is highly expressed in right-sided tumor cells and closely associated with circadian rhythms. NONO is a critical component of the mammalian circadian rhythm transcription-translation feedback loop. Previous studies indicate that BMAL1:CLOCK initiates the core negative feedback (Per/Cry) via E-box–mediated transcription, while NONO, functioning as a PER-associated factor, conveys circadian signals at defined genomic loci and counteracts its repressive activity [30]. NONO is highly expressed in various tumors and promotes proliferation, invasion, metastasis, and chemoresistance. In breast cancer, NONO promotes cancer cell proliferation by regulating the pre-mRNA splicing of genes related to cell proliferation, such as S-phase kinase-associated protein 2 and E2F transcription factor 8, and the expression level of NONO is significantly correlated with the prognosis of breast cancer patients [31]. Additionally, small-molecule compounds targeting NONO can inhibit the expression of androgen receptor and its splice variants in prostate cancer by covalently binding to the C145 site of NONO, thereby inhibiting cancer cell proliferation [32]. In glioblastoma, NONO cooperatively binds to the intron region of GPX1 pre-mRNA with PSPC1, regulating the alternative splicing of GPX1, which affects tumor growth, invasion, and redox homeostasis [24].

To further explore the potential role of NONO in the tumor microenvironment, we analyzed the communication relationships between NONO⁺ TC and other cells. Our study revealed a specific communication link between NONO⁺ TC and fibroblasts, primarily mediated by COLLAGEN, FN1, LAMININ, PDGF, TENASCIN, and THBS. Notably, TENASCIN and THBS signals were exclusively present in NONO-expressing tumor cells. TENASCIN (TN) and THBS (Thrombospondin) are two critical extracellular matrix proteins that play pivotal roles in the malignant tumor microenvironment. Tenascin-C (TNC) promotes tumor progression through multiple mechanisms within the tumor microenvironment. First, TNC binds to CXCL12, which retains CD8^+^ T cells in the stroma and limits cytotoxicity, thereby promoting breast cancer progression [33]. In pancreatic cancer, however, TNC is primarily expressed by CAFs and engages integrin receptors on tumor cells to promote EMT, enhance invasiveness, and confer drug resistance [34]. The Thrombospondin family exerts various biological functions in the tumor microenvironment, including regulating tumor cell proliferation, migration, angiogenesis, and immune evasion. THBS1 can modulate innate and adaptive immune cells through a CD47-dependent mechanism, restricting anti-tumor immune responses [35]. In hepatocellular carcinoma, cancer stem cells lacking THBS2 promote tumor invasiveness and drug resistance by regulating matrix metalloproteinase (MMP) activity and matrix stiffness [36]. Moreover, in early-stage lung adenocarcinoma, CAFs with high THBS2 expression promote tumor invasion and metastasis by inhibiting anti-tumor immunity and are associated with poor patient prognosis [37]. Further communication analysis stratified by fibroblast subtypes revealed that NONO⁺ TC receive a substantial proportion of incoming signals from fibroblasts within the tumour microenvironment. This selective interaction may potentiate malignant features of the tumour niche. As key accomplices in tumour progression, myCAFs promote cancer invasion and metastasis by remodeling the extracellular matrix, secrete a variety of growth factors to stimulate tumour cell proliferation and angiogenesis, and induce epithelial–mesenchymal transition, thereby enhancing tumour heterogeneity and therapeutic resistance [38, 39]. Consistent with our preliminary experimental validation, NONO knockdown led to a marked decrease in the mRNA levels of the core clock gene PER2 and the downstream tumor-suppressor DEC1, providing initial mechanistic support for our population-level observation that high NONO expression coincides with more pronounced circadian disruption. At the cell-extrinsic level, NONO knockdown significantly reduced transcription of key tumor-cell surface receptors—including ITGB1, SDC1, and CD47—thereby diminishing the cells’ capacity to receive fibroblast/matrix-derived cues. Taken together, these data suggest that in right-sided CRC, NONO promotes malignancy by simultaneously perturbing intrinsic clock output, which destabilizes proliferative control, and maintaining a receptor repertoire that heightens responsiveness to pro-tumor microenvironmental signals—thus tightly coupling circadian dysregulation with the molding of a malignant niche(Fig. 8).

In this study, we further developed a circadian rhythm–related gene signature for prognostication in CRC. Using LASSO regression, four key prognostic genes were identified: NOS2, PPARGC1A, DRD4, and NGFR. NOS2 expression is under the direct control of the core clock proteins CLOCK/BMAL1 and displays robust circadian oscillation [40]. In CRC, high NOS2 levels are strongly associated with tumour invasion, metastasis, and resistance to chemotherapy, and serve as an independent adverse predictor of both overall and recurrence-free survival [41]. PPARGC1A (PGC-1α), a pivotal integrator of metabolism and circadian timing, drives rhythmic transcription of core clock genes such as BMAL1 [42]. In CRC, elevated PGC-1α promotes glucose uptake and lipid synthesis to fuel rapid cell proliferation and increases oxidative metabolism, thereby fostering chemoresistance [43]; its over-expression correlates with advanced lymph-node stage and poorer prognosis, making it a strong indicator of shortened survival [42]. These findings underscore PGC-1α’s role in circadian-disruption-driven tumour progression. DRD4, a key dopamine receptor, relays light–dark signals in the retina to synchronise intracellular clocks [44]; in CRC, aberrant DRD4 up-regulation activates the TGF-β pathway, enhancing tumour migration and invasion [45]. NGFR modulates feeding-related rhythmic behaviour in the hypothalamus and AgRP neurons, thereby linking energy balance with circadian control [46]; in CRC, NGFR is frequently silenced by promoter methylation, and its loss is associated with increased proliferation and poor prognosis [47]. Collectively, these genes may influence patient outcomes by reshaping circadian mechanisms within tumor cells. We first evaluated the association between circadian disruption and prognosis in the TCGA-CRC cohort, confirming a strong link between rhythm dysregulation and adverse outcomes. However, in external GEO validation sets, although survival curves remained separated, time-dependent ROC analyses showed lower AUCs than in TCGA, indicating reduced discriminatory performance in external datasets. This discrepancy may reflect batch effects across sequencing platforms, differences in clinical baselines, and molecular-subtype heterogeneity. Therefore, future work should recalibrate and optimise this model in larger, multi-centre prospective cohorts to facilitate its translation into a clinically actionable stratification tool. In conclusion, the prognostic model based on circadian rhythm-related genes exhibits promising potential for application in colorectal cancer patients. However, due to the limited sample size, these findings need to be validated in larger, independent cohorts. Further functional studies are also necessary to better understand the biological roles of these genes in colorectal cancer.

Although this study systematically delineates the association between NONO, circadian-rhythm disruption, and microenvironmental remodeling in right-sided colorectal cancer using multi-omics analyses and preliminary functional assays, several limitations remain. First, our core mechanistic proposition—that NONO enhances tumor-cell reception of fibroblast-derived cues—relies on bioinformatic inference; future work should test the dynamics of this communication axis in more physiological settings. Second, we did not perform synchronized, multi–time-point clock assays, which limits mechanistic inference regarding circadian control; accordingly, a dedicated follow-up study will employ dexamethasone/serum-shock synchronization with time-series profiling of the core clock network, combined with NONO perturbation–rescue experiments, to define NONO’s causal role in circadian-rhythm phenotypes. Third, in vivo evidence directly linking NONO to tumor-microenvironment remodeling and malignant progression in right-sided CRC is lacking; therefore, xenograft or orthotopic models will be used to evaluate how modulating NONO affects tumor growth, matrix remodeling, and interactions with the myCAF compartment, together with longitudinal circadian assessments to confirm the positive association between NONO expression and circadian-rhythm disruption.

## Conclusion

Our study delineates a circadian-regulated, NONO-centric tumor–stroma interaction that underlies left–right heterogeneity in colorectal cancer. The right-sided CRC is characterized by more pronounced circadian rhythm disruption, a phenotype closely linked to the positive expression of the core circadian factor NONO. Interestingly, our research reveals a significant pro-tumorigenic role of NONO. NONO does not directly drive signal output; rather, it transforms tumor cells into “receivers” that are highly responsive to signals from fibroblasts, especially the pro-tumorigenic myCAF subtype, by increasing the expression of essential receptors. This discovery establishes a complete chain of evidence from intrinsic rhythm disruption to extrinsic microenvironment remodeling. This positions NONO as a critical signaling hub that links the cell-autonomous clock to tumor-stroma crosstalk (Fig. 9).

## Supplementary Information

Below is the link to the electronic supplementary material.

Supplementary Material 1

## Data Availability

The data that support the findings of this study are available from the corresponding author upon reasonable request.

## References

[1] Fagiani F, Di Marino D, Romagnoli A, Travelli C, Voltan D, Di Cesare ML, et al. Molecular regulations of circadian rhythm and implications for physiology and diseases. Signal Transduct Target Ther. 2022;7(1):41. 35136018 10.1038/s41392-022-00899-y PMC8825842

[2] Flanagan A, Bechtold DA, Pot GK, Johnston JD. Chrono-nutrition: from molecular and neuronal mechanisms to human epidemiology and timed feeding patterns. J Neurochem. 2021;157(1):53–72. 33222161 10.1111/jnc.15246

[3] Fishbein AB, Knutson KL, Zee PC. Circadian disruption and human health. J Clin Invest. 2021;131(19). 10.1172/JCI148286 PMC8483747 34596053

[4] Boivin DB, Boudreau P, Kosmadopoulos A. Disturbance of the circadian system in shift work and its health impact. J Biol Rhythms. 2022;37(1):3–28. 34969316 10.1177/07487304211064218 PMC8832572

[5] Yang Z, Black K, Ohman-Strickland P, Graber JM, Kipen HM, Fang M, et al. Disruption of central and peripheral circadian clocks and circadian controlled Estrogen receptor rhythms in night shift nurses in working environments. FASEB J. 2024;38(11):e23719. 38837828 10.1096/fj.202302261RR PMC11884403

[6] Brown SA, Ripperger J, Kadener S, Fleury-Olela F, Vilbois F, Rosbash M, et al. PERIOD1-associated proteins modulate the negative limb of the mammalian circadian oscillator. Science. 2005;308(5722):693–6. 15860628 10.1126/science.1107373

[7] Benegiamo G, Mure LS, Erikson G, Le HD, Moriggi E, Brown SA, et al. The RNA-binding protein NONO coordinates hepatic adaptation to feeding. Cell Metab. 2018;27(2):404–18. 29358041 10.1016/j.cmet.2017.12.010 PMC6996513

[8] Papagiannakopoulos T, Bauer MR, Davidson SM, Heimann M, Subbaraj L, Bhutkar A, et al. Circadian rhythm disruption promotes lung tumorigenesis. Cell Metab. 2016;24(2):324–31. 27476975 10.1016/j.cmet.2016.07.001 PMC5367626

[9] Qu M, Zhang G, Qu H, Vu A, Wu R, Tsukamoto H, et al. Circadian regulator BMAL1::CLOCK promotes cell proliferation in hepatocellular carcinoma by controlling apoptosis and cell cycle. Proc Natl Acad Sci U S A. 2023;120(2):e2214829120. 36595671 10.1073/pnas.2214829120 PMC9926257

[10] Li M, Hao B, Zhang M, Reiter RJ, Lin S, Zheng T, et al. Melatonin enhances radiofrequency-induced NK antitumor immunity, causing cancer metabolism reprogramming and Inhibition of multiple pulmonary tumor development. Signal Transduct Target Ther. 2021;6(1):330. 34471091 10.1038/s41392-021-00745-7 PMC8410827

[11] Van Houten N, Blake SF, Li EJ, Hallam TA, Chilton DG, Gourley WK, et al. Elevated expression of bcl-2 and bcl-x by intestinal intraepithelial lymphocytes: resistance to apoptosis by glucocorticoids and irradiation. Int Immunol. 1997;9(7):945–53. 9237103 10.1093/intimm/9.7.945

[12] Shen H, Yang J, Huang Q, Jiang MJ, Tan YN, Fu JF, et al. Different treatment strategies and molecular features between right-sided and left-sided colon cancers. World J Gastroenterol. 2015;21(21):6470–8. 26074686 10.3748/wjg.v21.i21.6470 PMC4458758

[13] Guo JN, Chen D, Deng SH, Huang JR, Song JX, Li XY, et al. Identification and quantification of immune infiltration landscape on therapy and prognosis in left- and right-sided colon cancer. Cancer Immunol Immunother. 2022;71(6):1313–30. 34657172 10.1007/s00262-021-03076-2 PMC9122887

[14] Hsu YL, Lin CC, Jiang JK, Lin HH, Lan YT, Wang HS, et al. Clinicopathological and molecular differences in colorectal cancer according to location. Int J Biol Markers. 2019;34(1):47–53. 30854932 10.1177/1724600818807164

[15] Zhu S, Tu J, Pei W, Zheng Z, Bi J, Feng Q. Development and validation of prognostic nomograms for early-onset colon cancer in different tumor locations: a population-based study. BMC Gastroenterol. 2023;23(1):362. 37865754 10.1186/s12876-023-02991-1 PMC10590526

[16] Shang Z, Xi S, Lai Y, Cheng H. Single-cell transcriptomics and Mendelian randomization reveal LUCAT1’s role in right-sided colorectal cancer risk. Front Genet. 2024;15:1357704. 38711918 10.3389/fgene.2024.1357704 PMC11070547

[17] Huang X, Liu J, Mo X, Liu H, Wei C, Huang L, et al. Systematic profiling of alternative splicing events and splicing factors in left- and right-sided colon cancer. Aging. 2019;11(19):8270–93. 31586988 10.18632/aging.102319 PMC6814588

[18] Chen Y, Song J, Ruan Q, Zeng X, Wu L, Cai L, et al. Single-cell sequencing methodologies: from transcriptome to multi-dimensional measurement. Small Methods. 2021;5(6):e2100111. 34927917 10.1002/smtd.202100111

[19] Zormpas E, Queen R, Comber A, Cockell SJ. Mapping the transcriptome: realizing the full potential of Spatial data analysis. Cell. 2023;186(26):5677–89. 38065099 10.1016/j.cell.2023.11.003

[20] Rao A, Barkley D, Franca GS, Yanai I. Exploring tissue architecture using Spatial transcriptomics. Nature. 2021;596(7871):211–20. 34381231 10.1038/s41586-021-03634-9 PMC8475179

[21] Zhou L, Zhang Z, Nice E, Huang C, Zhang W, Tang Y. Circadian rhythms and cancers: the intrinsic links and therapeutic potentials. J Hematol Oncol. 2022;15(1):21. 35246220 10.1186/s13045-022-01238-y PMC8896306

[22] Shafi AA, Knudsen KE. Cancer and the circadian clock. Cancer Res. 2019;79(15):3806–14. 31300477 10.1158/0008-5472.CAN-19-0566 PMC8121183

[23] Zhao G, Liu R, Ge L, Qi D, Wu Q, Lin Z, et al. NONO regulates m(5)c modification and alternative splicing of PTEN mRNAs to drive gastric cancer progression. J Exp Clin Cancer Res. 2025;44(1):81. 40033337 10.1186/s13046-024-03260-z PMC11877715

[24] Wang X, Han M, Wang S, Sun Y, Zhao W, Xue Z, et al. Targeting the splicing factor NONO inhibits GBM progression through GPX1 intron retention. Theranostics. 2022;12(12):5451–69. 35910786 10.7150/thno.72248 PMC9330516

[25] Shen M, Zhang R, Jia W, Zhu Z, Zhao X, Zhao L, et al. Nuclear scaffold protein p54(nrb)/NONO facilitates the hypoxia-enhanced progression of hepatocellular carcinoma. Oncogene. 2021;40(24):4167–83. 34079086 10.1038/s41388-021-01848-9 PMC8211563

[26] Lau KS, Dennis JW. N-glycans in cancer progression. Glycobiology. 2008;18(10):750–60. 18701722 10.1093/glycob/cwn071

[27] Kizuka Y, Taniguchi N. Enzymes for n-glycan branching and their genetic and nongenetic regulation in cancer. Biomolecules. 2016;6(2). 10.3390/biom6020025 PMC4919920 27136596

[28] Yin J, Ren W, Huang X, Deng J, Li T, Yin Y. Potential mechanisms connecting purine metabolism and cancer therapy. Front Immunol. 2018;9:1697. 30105018 10.3389/fimmu.2018.01697 PMC6077182

[29] Bonifacio VDB, Pereira SA, Serpa J, Vicente JB. Cysteine metabolic circuitries: druggable targets in cancer. Br J Cancer. 2021;124(5):862–79. 33223534 10.1038/s41416-020-01156-1 PMC7921671

[30] Kowalska E, Ripperger JA, Hoegger DC, Bruegger P, Buch T, Birchler T, et al. NONO couples the circadian clock to the cell cycle. Proc Natl Acad Sci U S A. 2013;110(5):1592–9. 23267082 10.1073/pnas.1213317110 PMC3562797

[31] Iino K, Mitobe Y, Ikeda K, Takayama KI, Suzuki T, Kawabata H, et al. RNA-binding protein NONO promotes breast cancer proliferation by post-transcriptional regulation of SKP2 and e2f8. Cancer Sci. 2020;111(1):148–59. 31733123 10.1111/cas.14240 PMC6942431

[32] Kathman SG, Koo SJ, Lindsey GL, Her HL, Blue SM, Li H, et al. Remodeling oncogenic transcriptomes by small molecules targeting NONO. Nat Chem Biol. 2023;19(7):825–36. 36864190 10.1038/s41589-023-01270-0 PMC10337234

[33] Murdamoothoo D, Sun Z, Yilmaz A, Riegel G, Abou-Faycal C, Deligne C, et al. Tenascin-c immobilizes infiltrating t lymphocytes through CXCL12 promoting breast cancer progression. EMBO Mol Med. 2021;13(6):e13270. 33988305 10.15252/emmm.202013270 PMC8185552

[34] Furuhashi S, Morita Y, Matsumoto A, Ida S, Muraki R, Kitajima R, et al. Tenascin c in pancreatic cancer-associated fibroblasts enhances epithelial mesenchymal transition and is associated with resistance to immune checkpoint inhibitor. Am J Cancer Res. 2023;13(11):5641–55. 38058842 PMC10695794

[35] Kaur S, Bronson SM, Pal-Nath D, Miller TW, Soto-Pantoja DR, Roberts DD. Functions of thrombospondin-1 in the tumor microenvironment. Int J Mol Sci. 2021;22(9). 10.3390/ijms22094570 PMC8123789 33925464

[36] Ng KY, Shea QT, Wong TL, Luk ST, Tong M, Lo CM, et al. Chemotherapy-enriched THBS2-deficient cancer stem cells drive hepatocarcinogenesis through matrix softness induced histone h3 modifications. Adv Sci (Weinh). 2021;8(5):2002483. 33717837 10.1002/advs.202002483 PMC7927606

[37] Yang H, Sun B, Fan L, Ma W, Xu K, Hall S, et al. Multi-scale integrative analyses identify THBS2(+) cancer-associated fibroblasts as a key orchestrator promoting aggressiveness in early-stage lung adenocarcinoma. Theranostics. 2022;12(7):3104–30. 35547750 10.7150/thno.69590 PMC9065207

[38] Kieffer Y, Hocine HR, Gentric G, Pelon F, Bernard C, Bourachot B, et al. Single-cell analysis reveals fibroblast clusters linked to immunotherapy resistance in cancer. Cancer Discov. 2020;10(9):1330–51. 32434947 10.1158/2159-8290.CD-19-1384

[39] Qi J, Sun H, Zhang Y, Wang Z, Xun Z, Li Z, et al. Single-cell and Spatial analysis reveal interaction of FAP(+) fibroblasts and SPP1(+) macrophages in colorectal cancer. Nat Commun. 2022;13(1):1742. 35365629 10.1038/s41467-022-29366-6 PMC8976074

[40] Liao W, Ye T, Liu H. Prognostic value of inducible nitric oxide synthase (iNOS) in human cancer: a systematic review and meta-analysis. Biomed Res Int. 2019;2019:6304851. 31275981 10.1155/2019/6304851 PMC6582868

[41] Zafirellis K, Zachaki A, Agrogiannis G, Gravani K. Inducible nitric oxide synthase expression and its prognostic significance in colorectal cancer. APMIS. 2010;118(2):115–24. 20132175 10.1111/j.1600-0463.2009.02569.x

[42] Liu C, Li S, Liu T, Borjigin J, Lin JD. Transcriptional coactivator PGC-1alpha integrates the mammalian clock and energy metabolism. Nature. 2007;447(7143):477–81. 17476214 10.1038/nature05767

[43] Wang Y, Peng J, Yang D, Xing Z, Jiang B, Ding X, et al. From metabolism to malignancy: the multifaceted role of PGC1alpha in cancer. Front Oncol. 2024;14:1383809. 38774408 10.3389/fonc.2024.1383809 PMC11106418

[44] Jackson CR, Chaurasia SS, Hwang CK, Iuvone PM. Dopamine d(4) receptor activation controls circadian timing of the adenylyl cyclase 1/cyclic AMP signaling system in mouse retina. Eur J Neurosci. 2011;34(1):57–64. 21676039 10.1111/j.1460-9568.2011.07734.x PMC3129439

[45] Zhou Y, Tang J, Weng M, Zhang H, Lai M. DRD4 interacts with TGF-beta receptors to drive colorectal cancer metastasis independently of dopamine signaling pathway. Adv Sci (Weinh). 2025;12(6):e2413953. 39679842 10.1002/advs.202413953 PMC11809390

[46] Podyma B, Johnson D, Sipe L, Remcho TP, Battin K, Liu Y et al. The p75 neurotrophin receptor in AgRP neurons is necessary for homeostatic feeding and food anticipation. Elife. 2020;9(. 10.7554/eLife.52623 PMC7056271 31995032

[47] Yang Z, Chen H, Huo L, Yang Z, Bai Y, Fan X, et al. Epigenetic inactivation and tumor-suppressor behavior of NGFR in human colorectal cancer. Mol Cancer Res. 2015;13(1):107–19. 25244921 10.1158/1541-7786.MCR-13-0247

