# Supplementary material for: NONO links circadian rhythm disruption and enhanced tumor-fibroblast crosstalk in right-sided colorectal cancer
Source: Biomark Res. 2025 Oct 31;13:138. doi: 10.1186/s40364-025-00852-5 (PMC12577322; doi:10.1186/s40364-025-00852-5)
Supplement: Supplementary file 1 — Supplementary Material 1 [file 40364_2025_852_MOESM1_ESM.docx]

**Supplementary materials**

**sFig1. Single cell dimensionality reduction clustering and cell subpopulation identification.** **(A)** Principal Component Analysis (PCA) of Different Cell Populations. Each point represents an individual cell, colored according to its cell cluster (T_cas1 to T_cas9). PC_1 and PC_2 are the first two principal components, capturing the main variance in the dataset. **(B)** Expression Levels of Marker Genes in Different Cells. The dot plot shows genes (rows) and cells (columns). The color intensity indicates the expression level of each gene. **(C)** Top 5 Expression Profiles of Each Cell Population. The heatmap displays genes (rows) and cell subpopulations (columns). Color intensity represents the average expression level of each gene in the corresponding cell cluster, as indicated by the legend. The cell types of each cluster are annotated on the right.


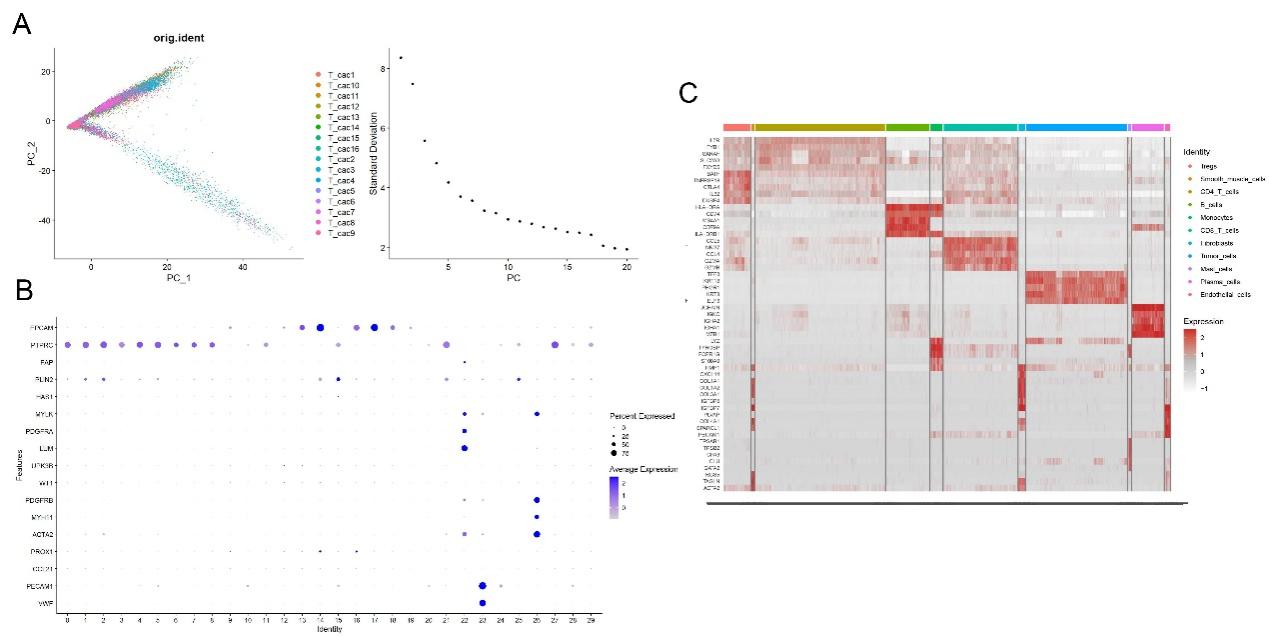


**sFig2. Spatial Transcriptomics Sample and Quality Control. (A-D)** Left Panel: HE-stained tissue section images of the samples. Middle Left Panel: UMAP dimensionality reduction clustering plot showing the distribution of different cell populations, with colors representing different cell clusters. Middle Right Panel: Violin plot of mitochondrial gene expression percentage (percent.mt), illustrating the distribution of mitochondrial gene expression across different cell populations. Right Panel: Spatial expression plots of nCount_Spatial and nFeature_Spatial in the samples, with color intensity indicating the counts' levels.


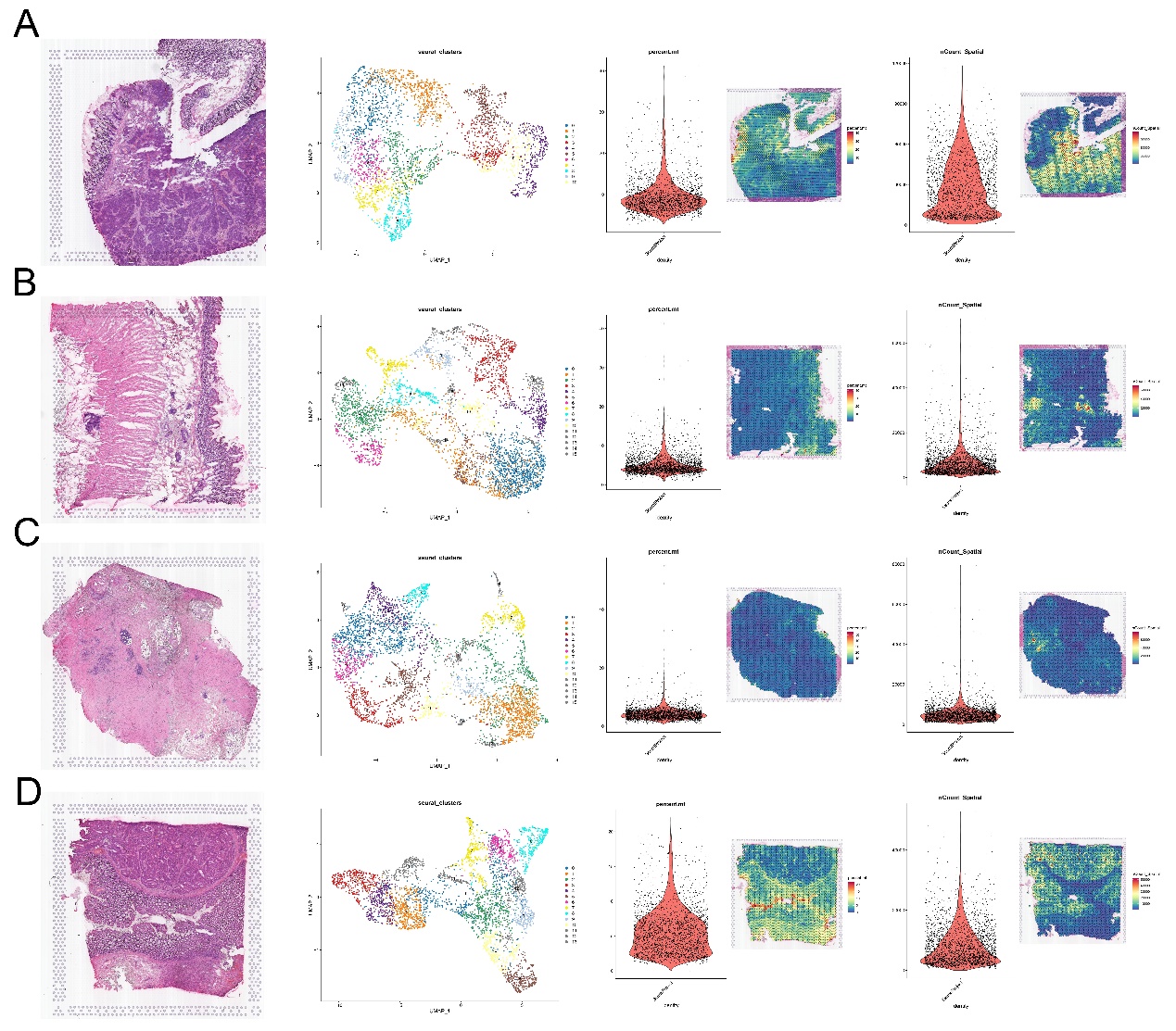


**sFig3. Cell-Cell Communication Analysis.** **(A)** Displays the communication networks between fibroblasts, NONO^-^ TC, and NONO^+^ TC with other cell types. Each node represents a cell type, with the node size proportional to the number of that cell type. The thickness of the edges indicates the strength of communication between cells, and the arrow direction shows the direction of signal transmission. **(B and C)** Dot plots showing the communication probabilities between NONO^-^ TC and other cell types, and between NONO^+^ TC and other cell types, respectively. Each dot represents a specific ligand-receptor pair, with the dot size indicating the p-value and color intensity representing the communication probability.


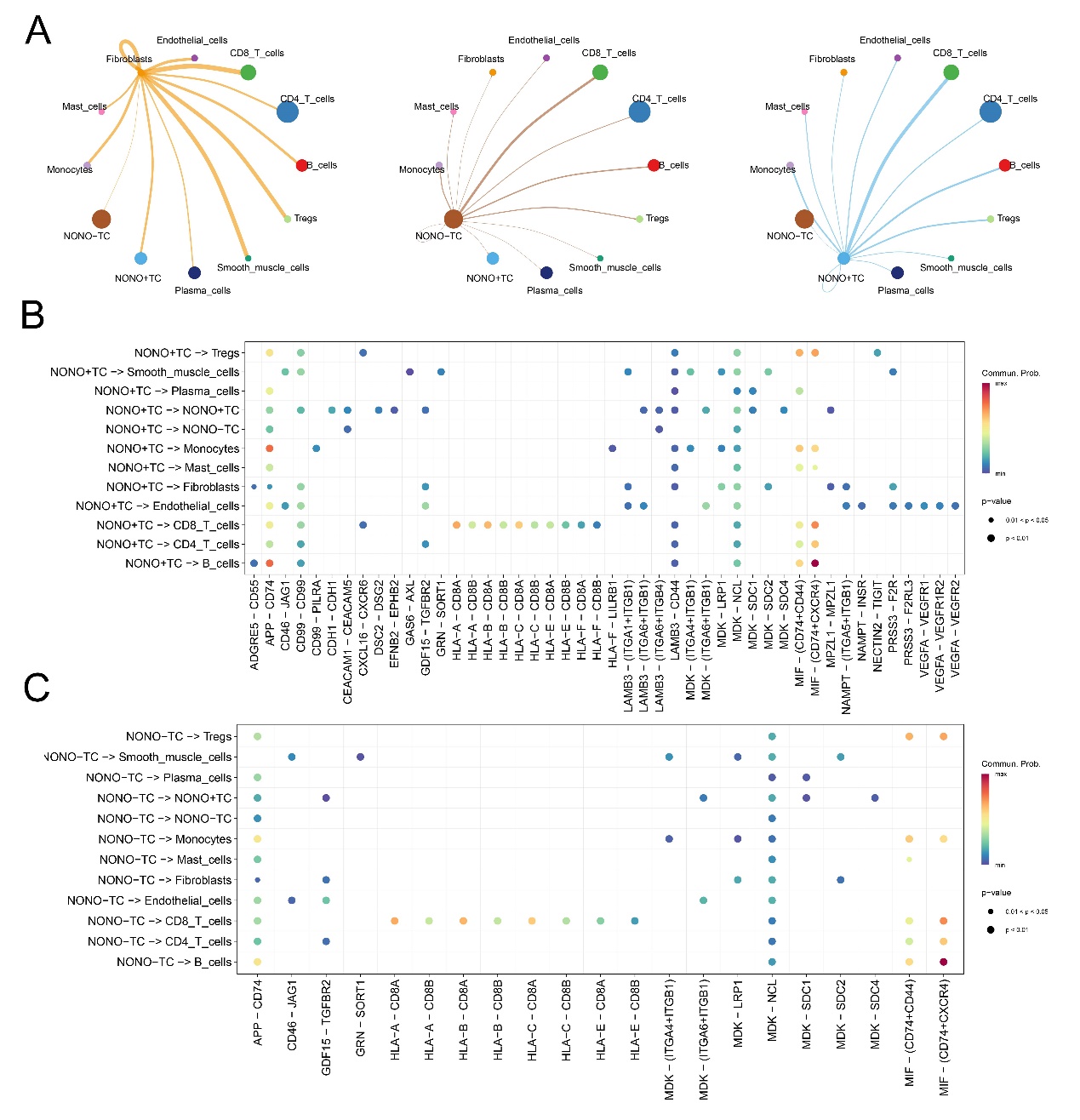


**sFig4. Distribution of Fibroblast Subtypes in Left- and Right-Sided Colorectal Cancer and Their Interactions with NONO^+^ TC.** (A) UMAP dimensionality reduction plot illustrating the distribution of fibroblast subtypes across left-sided (Left) and right-sided (Right) colorectal cancer. Colors denote distinct subtypes: apCAF (green), myCAF (red), and iCAF (blue). The distribution appears relatively uniform on the left, whereas myCAF and iCAF are more concentrated on the right. (B) Network diagram depicting interactions between fibroblast subtypes (iCAF, myCAF, apCAF) and NONO^+^ TC as well as NONO^-^ TC. The left panel shows the number of interactions, while the right panel indicates interaction weights/strength. Node size is proportional to the number of interactions, and edge thickness reflects interaction strength. Interactions between NONO^+^ TC and myCAF are notably stronger. (C) Heatmap illustrating the number of interactions and interaction weights/strength between signal sources (senders) and receivers. Color gradients range from light to dark, representing low to high values (red indicating high interaction numbers, blue indicating high interaction strength). myCAF emerges as the primary source, with significant interactions with NONO^+^ TC. (D) Scatter plot displaying the incoming and outgoing interaction strengths for each cell type. The x-axis represents outgoing interaction strength, the y-axis represents incoming interaction strength, and point size indicates interaction count. Colors correspond to subtypes: myCAF (green), apCAF (red), iCAF (blue). myCAF exhibits the highest interaction strength.


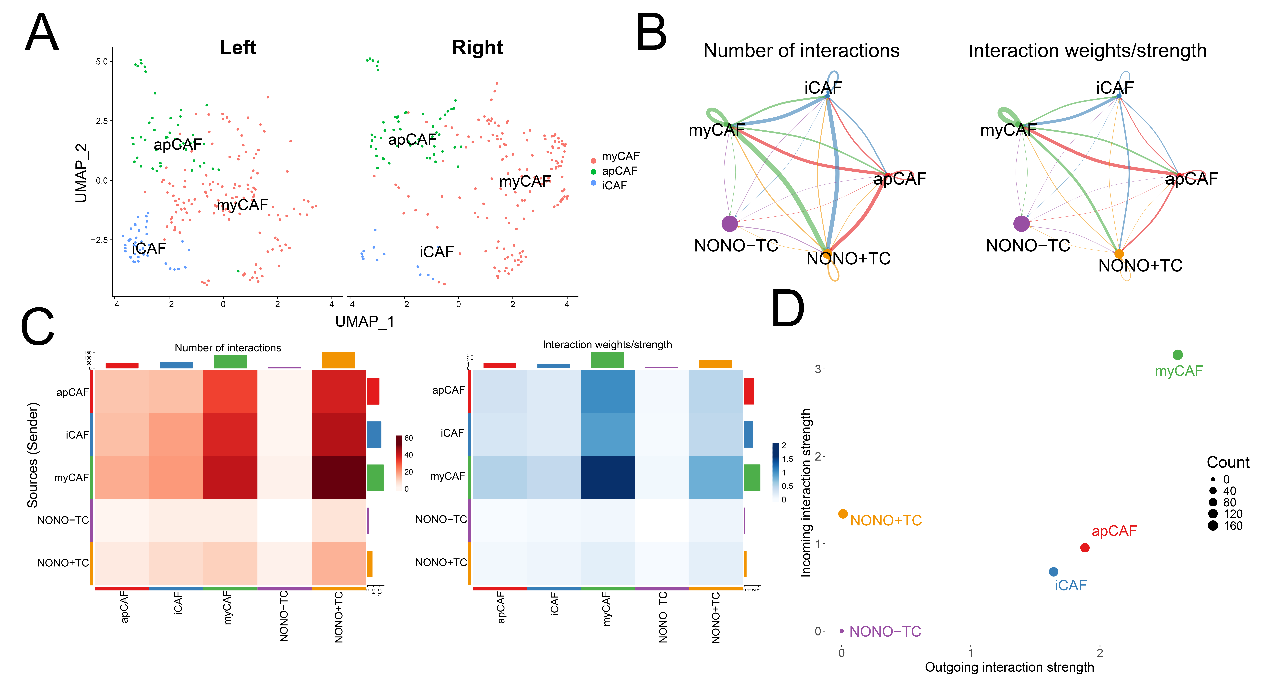


**sFig5. Batch-correction results for TCGA and GEO colorectal cancer gene-expression datasets.** (A) Principal-component analysis (PCA) of the merged TCGA‐CRC (blue) and GEO‐CRC (orange) samples before batch correction reveals a clear separation along PC1, indicating a strong study-specific effect. (B) PCA of the same data after ComBat adjustment (limma + sva pipeline) shows the two cohorts largely overlapping, demonstrating successful removal of the technical batch while preserving biological variance. Percentages on the axes indicate the variance explained by each component.


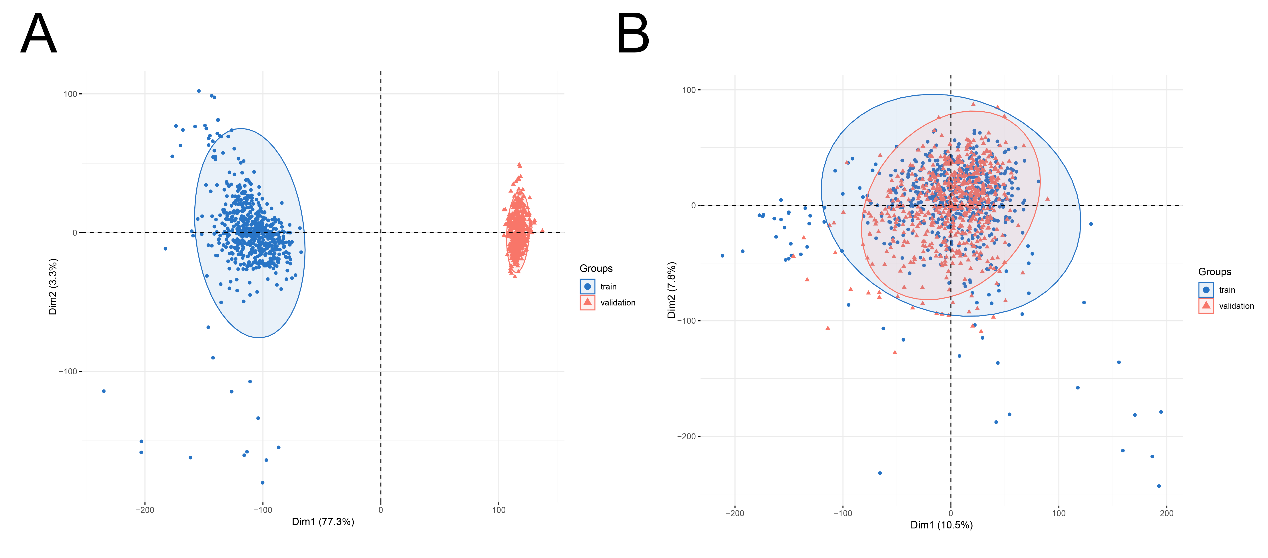


**sFig6. Construction of the Prognostic Model Based on Circadian Rhythm-Related Genes in Colorectal Cancer.** **(A)** LASSO coefficient profile of genes associated with the prognosis of colorectal cancer. **(B)** LASSO model cross-validation curve for selecting the optimal tuning parameter. The optimal lambda value is chosen based on the minimum criterion. **(C)** Forest plot displaying the hazard ratios and their 95% confidence intervals for the four genes included in the prognostic model. NOS2 and PPARGC1A are associated with reduced risk, whereas DRD4 and NGFR are linked to increased risk. **(D)** Kaplan-Meier survival curves for high-risk and low-risk groups based on the circadian rhythm-related gene prognostic model. Patients in the high-risk group show significantly poorer survival outcomes (p < 0.0001). **(E)** Time-dependent ROC curves evaluating the predictive accuracy of the four-gene model at 1 year, 2 years, and 5 years. The AUC values range from 0.703 to 0.716. **(F)** External validation cohort: the same four-gene signature stratifies patients into high- and low-risk groups with significantly different survival (log-rank p = 0.0069). **(G)** ROC analysis in the GSE39582 cohort yields AUCs of 0.56, 0.58 and 0.55 for 1-, 3- and 5-year survival, supporting moderate but consistent predictive performance in an independent dataset.
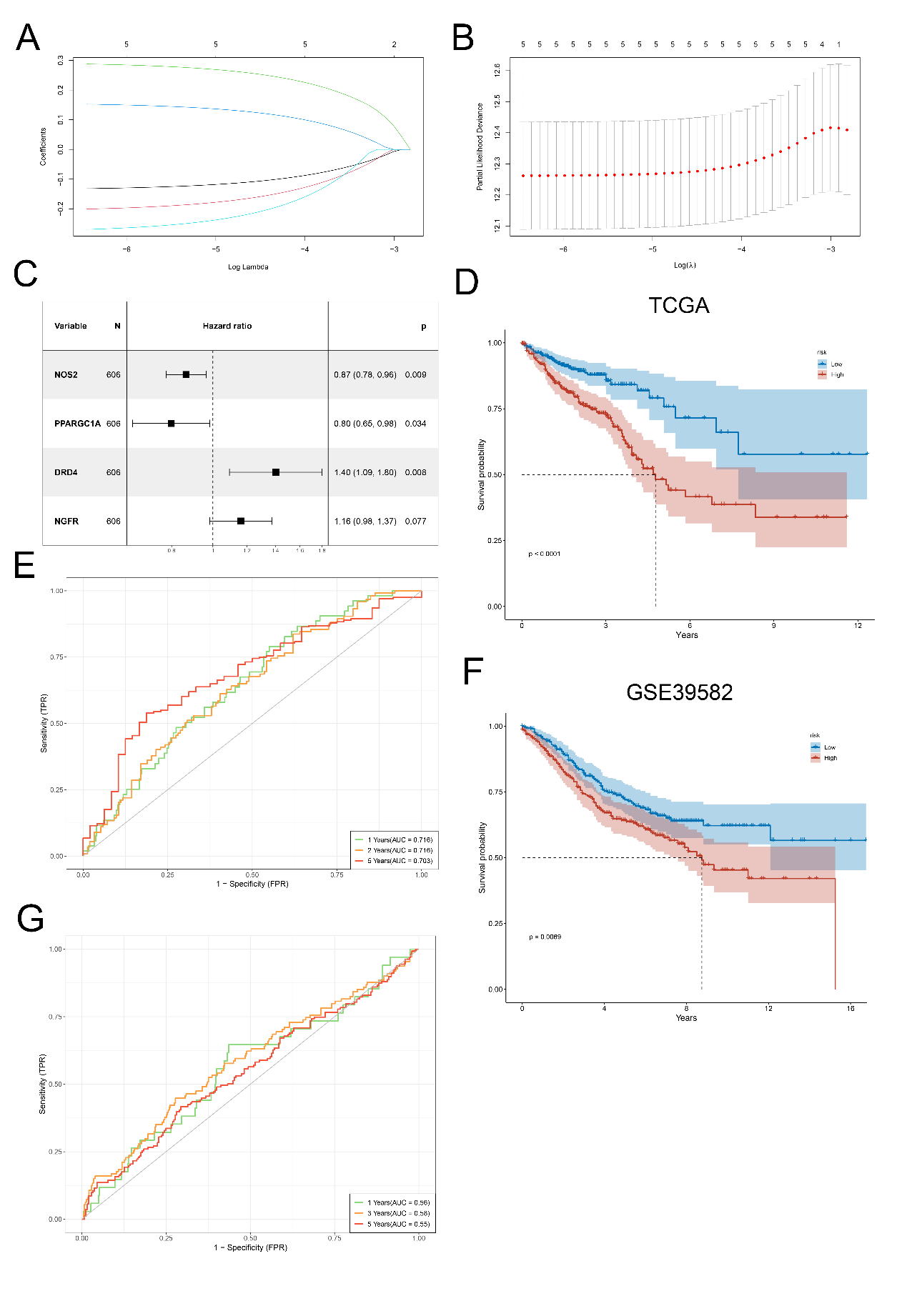


**sFig7. NONO knockdown efficiency in HCT116 and RKO cells.**Relative NONO mRNA expression in cells transfected with negative control siRNA (siNC) or three independent siRNAs targeting NONO (siNONO-1, -2, -3), measured by RT–qPCR and normalized to GAPDH. Data represent mean ± s.d. (n = 3 independent experiments). P < 0.01 (**), P < 0.001 (***), one-way ANOVA with Tukey’s test.

**
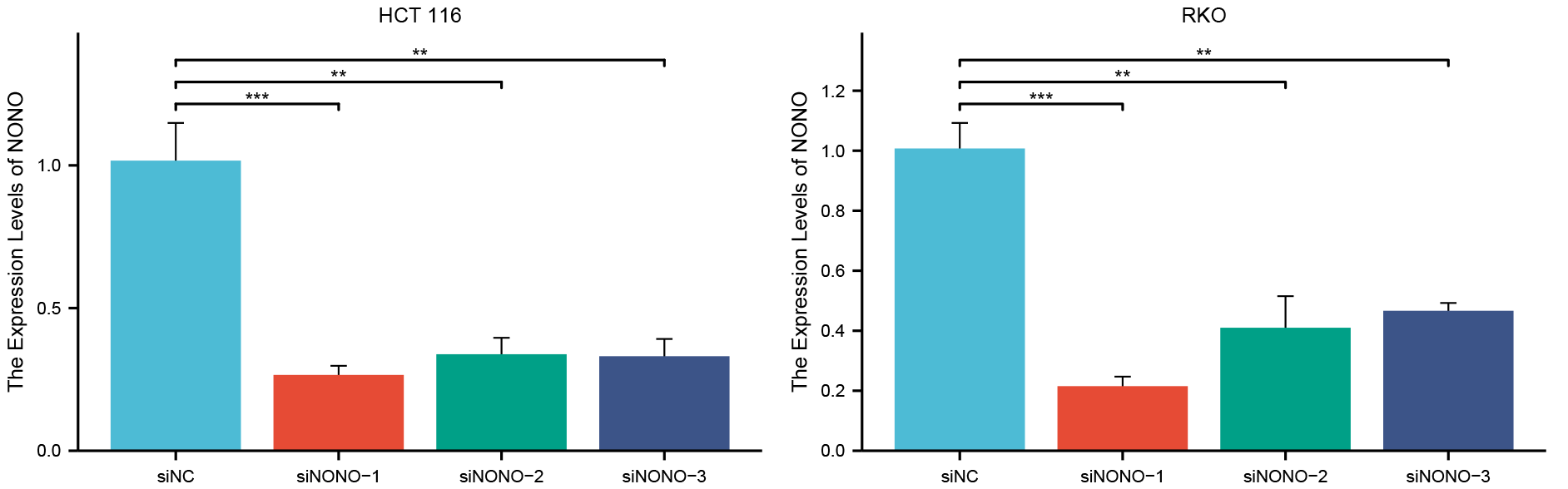
**

**sFig8. Ridgeline distributions of CRD scores stratified by tumor laterality and NONO status in tumor cells.**

Ridgeline (joy) plots display the distributions of CRD scores for left-sided and right-sided CRC, each further divided into NONO^+^ TC and NONO^-^ TC. In both laterality groups, the NONO^+^ TC distributions are consistently shifted toward higher CRD values relative to NONO^-^ TC, indicating greater circadian dysregulation in tumors with detectable NONO expression. Higher CRD scores denote more pronounced disruption of circadian rhythmicity.

**
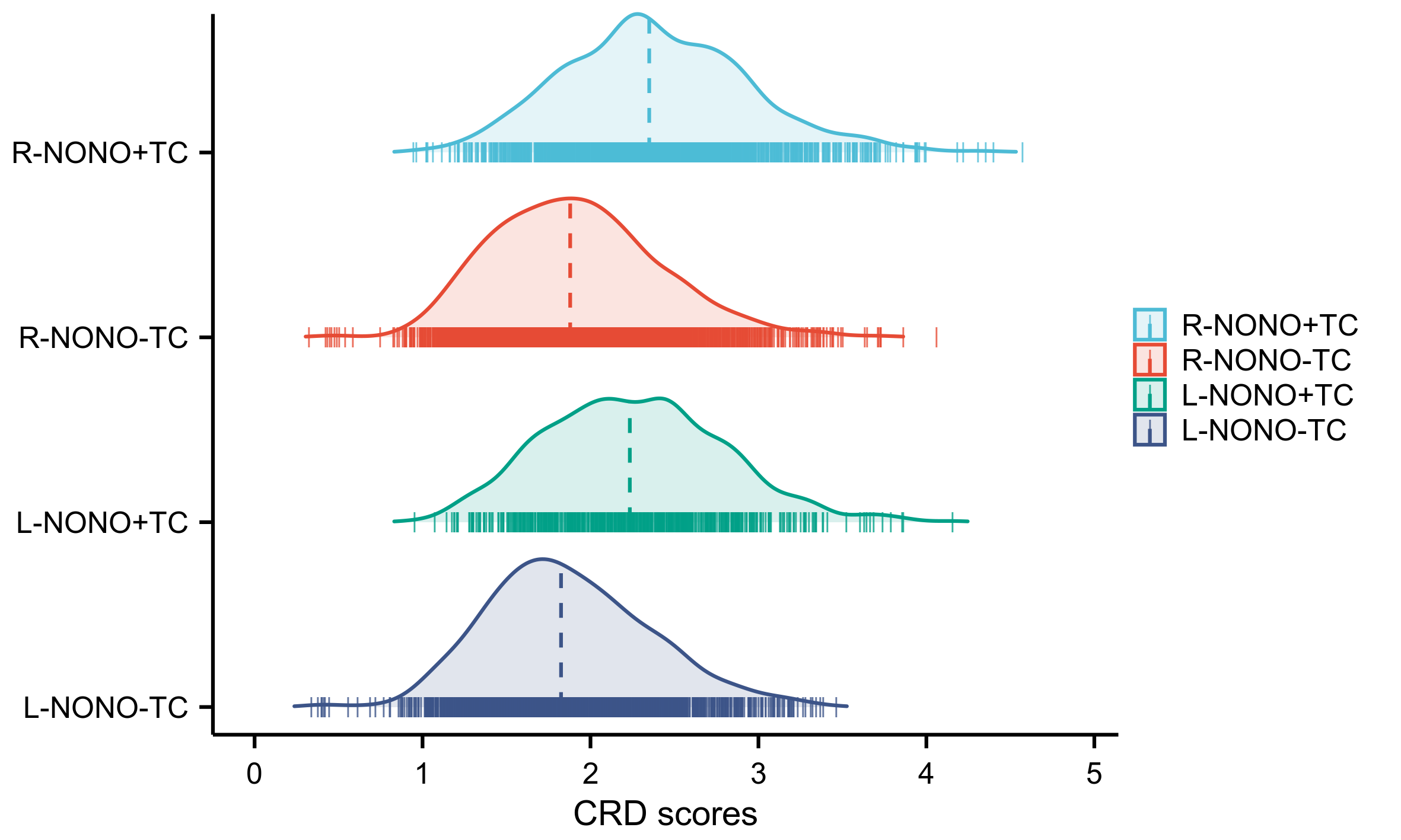
**

**Supplementary table 1. Coefficients of mistyR Model Views for Cell Types**

| **Target** | **Intercept** | **Intra** | **Juxta_5** | **p.intra** | **p.juxta_5** |
| --- | --- | --- | --- | --- | --- |
| NONO^+^ TC | -0.0253 | 0.8846 | -0.0075 | 0.0000 | 0.6464 |
| B.cells | -0.0037 | 0.5210 | 0.0509 | 0.0000 | 0.0223 |
| CD4.T.cells | -0.0054 | 0.6575 | 0.1511 | 0.0000 | 0.0000 |
| Fibroblasts | -0.0322 | 1.0327 | -0.2385 | 0.0000 | 0.0000 |
| Endothelial.cells | -0.0315 | 1.0346 | -0.2935 | 0.0000 | 0.0000 |
| CD8.T.cells | -0.0002 | 0.4814 | 0.1940 | 0.0000 | 0.0000 |
| Monocytes | -0.0107 | 0.6708 | 0.0465 | 0.0000 | 0.2014 |
| Mast.cells | -0.0004 | 0.2759 | 0.2946 | 0.0000 | 0.0000 |
| NONO^-^ TC | -0.0114 | 1.1352 | -0.0741 | 0.0000 | 0.0005 |
| Smooth.muscle.cells | -0.0134 | 0.9748 | -0.1426 | 0.0000 | 0.0000 |
| Tregs | -0.0040 | 0.7076 | 0.2384 | 0.0000 | 0.0000 |
| Plasma.cells | -0.0003 | 1.2774 | -0.0535 | 0.0000 | 0.1302 |

**Supplementary table 2. Importance of Fibroblasts in mistyR Model Views**

| **Target** | **Fibroblasts_intra_imp** | **Fibroblasts_juxta_5_imp** |
| --- | --- | --- |
| NONO^+^ TC | 10.424 | 8.806 |
| B.cells | 1.999 | 2.377 |
| CD4.T.cells | 3.315 | 3.291 |
| CD8.T.cells | 0.967 | 1.613 |
| Endothelial.cells | 4.979 | 4.436 |
| Mast.cells | 1.348 | 2.635 |
| Monocytes | 4.740 | 3.985 |
| NONO^-^ TC | 4.387 | 6.155 |
| Plasma.cells | 2.149 | 2.188 |
| Smooth.muscle.cells | 7.253 | 4.953 |
| Tregs | 1.480 | 2.179 |

**Supplementary table 3. Importance of NONO^+^TC in mistyR Model Views**

| **Target** | **NONO^+^TC_intra_imp** | **NONO^-^TC_juxta_5_imp** |
| --- | --- | --- |
| B.cells | 12.017 | 15.580 |
| CD4.T.cells | 14.520 | 14.250 |
| CD8.T.cells | 5.458 | 6.714 |
| Endothelial.cells | 36.279 | 32.251 |
| Fibroblasts | 55.214 | 40.041 |
| Mast.cells | 4.364 | 6.336 |
| Monocytes | 23.612 | 20.468 |
| NONO^-^ TC | 20.411 | 21.238 |
| Plasma.cells | 11.845 | 15.989 |
| Smooth.muscle.cells | 32.632 | 35.012 |
| Tregs | 4.805 | 11.702 |

**Supplementary table 4. Performance Metrics of mistyR Models**

| **Target** | **intra.RMSE** | **intra.R^2^** |  | **multi.RMSE** | **multi.R^2^** | **p.RMSE** | **p.R^2^** |
| --- | --- | --- | --- | --- | --- | --- | --- |
| NONO^+^ TC | 0.0721 | 90.1771 |  | 0.0685 | 91.1510 | 0.0223 | 0.0385 |
| B.cells | 0.0266 | 80.6366 |  | 0.0219 | 86.7097 | 0.0007 | 0.0068 |
| CD4.T.cells | 0.0386 | 65.6324 |  | 0.0368 | 68.6876 | 0.2533 | 0.2052 |
| Fibroblasts | 0.0445 | 81.0575 |  | 0.0430 | 82.2808 | 0.0519 | 0.0249 |
| Endothelial.cells | 0.0472 | 48.8990 |  | 0.0448 | 53.9726 | 0.1090 | 0.0085 |
| CD8.T.cells | 0.0123 | 7.2635 |  | 0.0121 | 9.6495 | 0.3376 | 0.0359 |
| Monocytes | 0.0235 | 43.9501 |  | 0.0225 | 48.7090 | 0.0480 | 0.0145 |
| Mast.cells | 0.0147 | 5.5428 |  | 0.0142 | 11.6249 | 0.2451 | 0.0001 |
| NONO^-^ TC | 0.0493 | 76.1549 |  | 0.0490 | 76.4818 | 0.4075 | 0.4394 |
| Smooth.muscle.cells | 0.0298 | 79.9522 |  | 0.0290 | 81.0273 | 0.1911 | 0.1599 |
| Tregs | 0.0198 | 25.2530 |  | 0.0191 | 29.4489 | 0.3160 | 0.1000 |
| Plasma.cells | 0.0024 | 38.9554 |  | 0.0024 | 39.5019 | 0.4970 | 0.4602 |

**Supplementary table 5. Nucleotide Sequences of Designed siRNAs for NONO Gene Silencing**

| Gene | Sequence(5'-3') | |
| --- | --- | --- |
| H4841-siNONO-1 | sense | GGACCAGUUAGAUGAUGAA(dT)(dT) |
|  | antisense | UUCAUCAUCUAACUGGUCC(dT)(dT) |
| H4841-siNONO-2 | sense | GGCUGUAGUCAUUGUGGAU(dT)(dT) |
|  | antisense | AUCCACAAUGACUACAGCC(dT)(dT) |
| H4841-siNONO-3 | sense | GGAAGAGCUGCACAACCAA(dT)(dT) |
|  | antisense | UUGGUUGUGCAGCUCUUCC(dT)(dT) |

**Supplementary table 6. Correlation Analysis Results of CRD Scores and NONO Expression**

|  | Mean | Standard Deviation  (SD) | Correlation Coefficient Type | Correlation Coefficient | P |
| --- | --- | --- | --- | --- | --- |
| CRD Scores | 2.073 | 0.567 |  |  |  |
| NONO  Expression | 0.485 | 0.782 |  |  |  |
| Correlation Results |  |  | Pearson | 0.262 | <0.001 |
|  |  |  | Spearman | 0.348 | <0.001 |
